# The polyol pathway and nuclear ketohexokinase A signaling drive hyperglycemia-induced metastasis of gastric cancer

**DOI:** 10.1038/s12276-023-01153-3

**Published:** 2024-01-10

**Authors:** Ye-Lim Kang, Jiyoung Kim, Su-Bin Kwak, Yi-Sook Kim, June Huh, Jong-Wan Park

**Affiliations:** 1 Department of Biomedical Science, BK21-Plus Education Program, Seoul National University College of Medicine, Daehak-ro, Jongno-gu, Seoul, 03080 Korea; 2 Department of Pharmacology, Seoul National University College of Medicine, Daehak-ro, Jongno-gu, Seoul, 03080 Korea; 3 Department of Chemical and Biological Engineering, Korea University, Anam-ro, Seongbuk-gu, Seoul, 02841 Korea; 4 Cancer Research Institute and Ischemic/Hypoxic Disease Institute, Seoul National University College of Medicine, Daehak-ro, Jongno-gu, Seoul, 03080 Korea

**Keywords:** Translational research, Gastric cancer

## Abstract

Diabetes might be associated with increased cancer risk, with several studies reporting hyperglycemia as a primary oncogenic stimulant. Since glucose metabolism is linked to numerous metabolic pathways, it is difficult to specify the mechanisms underlying hyperglycemia-induced cancer progression. Here, we focused on the polyol pathway, which is dramatically activated under hyperglycemia and causes diabetic complications. We investigated whether polyol pathway-derived fructose facilitates hyperglycemia-induced gastric cancer metastasis. We performed bioinformatics analysis of gastric cancer datasets and immunohistochemical analyses of gastric cancer specimens, followed by transcriptomic and proteomic analyses to evaluate phenotypic changes in gastric cancer cells. Consequently, we found a clinical association between the polyol pathway and gastric cancer progression. In gastric cancer cell lines, hyperglycemia enhanced cell migration and invasion, cytoskeletal rearrangement, and epithelial-mesenchymal transition (EMT). The hyperglycemia-induced acquisition of metastatic potential was mediated by increased fructose derived from the polyol pathway, which stimulated the nuclear ketohexokinase-A (KHK-A) signaling pathway, thereby inducing EMT by repressing the *CDH1* gene. In two different xenograft models of cancer metastasis, gastric cancers overexpressing AKR1B1 were found to be highly metastatic in diabetic mice, but these effects of AKR1B1 were attenuated by KHK-A knockdown. In conclusion, hyperglycemia induces fructose formation through the polyol pathway, which in turn stimulates the KHK-A signaling pathway, driving gastric cancer metastasis by inducing EMT. Thus, the polyol and KHK-A signaling pathways could be potential therapeutic targets to decrease the metastatic risk in gastric cancer patients with diabetes.

## Gastric cancer’s deadly dance with diabetes: the role of fructose in metastasis

Diabetes and cancer, two major worldwide health concerns, often coexist in patients. Recent research suggests diabetes can heighten the risk of various cancers. However, the precise reasons behind this link remain unknown. In this study, researchers discovered that high glucose levels (sugar in the blood), a diabetes hallmark, can enhance cancer cell aggression. This occurs via the polyol pathway (a process where glucose transforms into a substance named fructose). The fructose then triggers a specific signaling pathway (a series of chemical reactions) in the cancer cells, leading to their increased movement and invasion, signs of more aggressive cancers. This study offers a potential reason for the diabetes-cancer connection and implies that managing blood sugar levels in cancer patients with diabetes could be vital in preventing cancer progression. This summary was initially drafted using artificial intelligence, then revised and fact-checked by the author.

## Introduction

The incidence and prevalence of diabetes and cancer have been increasing worldwide. As of 2017, more than 450 million people have been diagnosed with diabetes^1^, and the incidence of cancer, excluding skin cancer, was ~20 million new cases per year^2^. Approximately 60% of newly diagnosed cancer patients are over the age of 65 years^3^, and ~25% of people over the age of 65 also have diabetes^4^. Thus, 8–18% of cancer patients also have diabetes in the overall population^5^, and diabetes is a frequent comorbidity of cancer patients in the elderly group over 65 years of age^6,7^. Recently, several clinical cohort studies have demonstrated that diabetes is a major risk factor for several cancers^8–10^. Moreover, studies have reported that diabetes is associated with the incidence, mortality, and progression of various cancers^6,11–13^. Experimental studies^14–16^ have revealed that hyperglycemia per se, rather than the common risk factors between diabetes and cancer, worsens cancer. Hyperglycemia renders cancer cells more aggressive by exerting metabolic and oxidative stresses^16–18^.

Glucose metabolism involves multiple intertwined metabolic pathways, dynamically generating numerous glucose metabolites, which affect cancer cell fate and behavior. For example, in the polyol pathway, aldo-keto reductase-1-member-1 (AKR1B1) reduces glucose to sorbitol, and sorbitol dehydrogenase (SORD) converts sorbitol to fructose. Normally, only ~3% of glucose is metabolized through the polyol pathway, but under hyperglycemia, over 30% of the glucose enters the pathway due to saturation of hexokinase action^19,20^. The polyol pathway is involved in the development of diabetic complications^21^. Under hyperglycemia, the polyol pathway depletes NADPH and generates excess NADH, resulting in a redox imbalance. Moreover, fructose generates advanced glycation end products (AGEs), facilitating oxidative stress. In addition, increased osmotic pressure due to sorbitol accumulation aggravates diabetic complications, such as neuropathy, nephropathy and retinopathy^21^. Several recent reports have demonstrated that the polyol pathway is involved in cancer progression and metastasis^22–24^. AKR1B1 has been reported to be overexpressed in several cancer types^25,26^. AKR1B1 inhibition suppresses cancer growth^24^, and its activation induces epithelial-mesenchymal transition (EMT) to facilitate cancer migration and invasion^22,23^. Nevertheless, little is known about the molecular mechanisms by which the polyol pathway promotes cancer progression.

Here, we investigated the role of the polyol pathway in the hyperglycemia-induced metastasis of gastric cancer. Hyperglycemia facilitated the migration and invasion of gastric cancer cells depending on the polyol pathway. In two xenograft models of gastric cancer, AKR1B1 overexpression increased metastasis in diabetic mice. Mechanistically, polyol pathway-derived fructose activated the KHK-A-YWHAH-SLUG pathway to induce EMT by repressing the *CDH1* gene. In conclusion, the polyol pathway promotes diabetes-induced gastric cancer metastasis by activating the KHK-A signaling pathway.

## Materials and methods

### Cell lines and cell culture

The human gastric cancer cell lines AGS, MKN-1, MKN-28, and MKN-45 were obtained from American Type Culture Collection (ATCC; Manassas, VA); SNU-216, SNU-484, SNU-601, and SNU-638 were obtained from the Korean Cell Line Bank (Seoul, South Korea). All cell lines except MKN-28 were cultured with 10% heat-inactivated fetal bovine serum (FBS) in RPMI and MKN-28 in DMEM. MKN-28 and SNU-638 gastric cancer cells were transfected with the CMV luciferase-IRES-GFP plasmid or the CMV luciferase-IRES-AKR1B1 plasmid and selected using G418 (Millipore, Burlington, MA). Five clones of stable cell lines were pooled to rule out artifacts by plasmid insertion into genomes. Cell lines were authenticated by STR DNA profiling analysis, which was performed in the Korean Cell Line Bank (Seoul, Korea). Mycoplasma contamination was routinely assessed when the cell growth or shape was changed. After thawing, the cells were usually cultured for no more than 3 months. Cells were grown in a humidified atmosphere containing 5% CO_2_ at 37 °C. For high glucose (HG) conditions, cells were incubated in RPMI and DMEM with 50 mM glucose.

### Materials

Culture media, bovine serum, epalrestat, streptozocin, G418, puromycin dihydrochloride and other chemicals were purchased from Sigma‒Aldrich (St. Louis, MO). A monoclonal antibody against S25-phosphorylated YWHAH was raised using phage display technology through a commercial facility (Bioneer, Korea). The antibodies used in this work are listed in Supplementary Table 1.

### Preparation of plasmids, siRNAs, and transfection

The cDNAs for human *AKR1B1*, *SORD*, *KHK-A*, and *YWHAH* were cloned by reverse transcription and PCR using Pfu DNA polymerase and inserted into pcDNA-Myc, pcDNA-His, or pcDNA-FLAG vectors. For transient expression and knockdown, cells at ~70% confluence were transfected using Jet-Prime reagent (Polyplus, Illkirch, France) and Lipofectamine RNAiMAX reagent (Invitrogen), respectively. Nucleotide sequences of siRNAs are summarized in Supplementary Table 2.

### Gastric cancer xenografts

All animal experiments were carried out with the proposed protocol approved by the Institutional Animal Care and Use Committee (approval no. SNU-190702-3-3; SNU-230327-5-1). MKN-28 and SNU-638 gastric cancer cell lines harboring the luciferase-IRES-GFP plasmid or the luciferase-IRES-AKR1B1 plasmid were selected using G418 (Millipore, Burlington, MA). The cells were secondarily transfected with sh-RNAs, and the stable cell lines were further selected using puromycin. Male 8-week-old BALB/cSlc-nu/nu mice were intravenously injected with streptozotocin (100 mg/kg) five times for 3 weeks. On the 10th week after the first streptozotocin injection, transfected MKN-28 and SNU-638 cells were implanted in the spleen and subcutaneously injected into the left flank of mice. After 4 weeks (MKN-28) or 10–15 weeks (SNU-638), mice were intraperitoneally injected with VivoGlo luciferin (Promega). Tumors were monitored using Xenogen IVIS100 and IVIS Spectrum with LivingImage 2.50.1 (Xenogen, Alameda, CA).

### Cell migration and invasion assays

For migration analysis, cells in serum-free RPMI were seeded onto the upper chamber with an 8.0 μm pore filter (Corning Life Science, Acton, MA), and the lower chamber was filled with 10% serum. For invasion analysis, a Matrigel-coated filter was used. After 18 h of incubation, cells on the lower surface of the filter were fixed, stained with H&E, photographed, and counted using ImageJ software (NIH, Bethesda, MD).

### Quantitative RT‒PCR

Total RNA was extracted using TRIzol reagent (Invitrogen) and reverse-transcribed using M-MLV Reverse Transcriptase (Enzynomics; Daejeon, Korea) at 42 °C for 60 min. Real-time PCR was performed in qPCR Mastermix (Enzynomics) using a CFX Connect Real-Time Cycler (Bio-Rad, Hercules, CA). The mRNA levels were calculated in reference to the GAPDH level. All reactions were performed in triplicate. The nucleotide sequences of the PCR primers are summarized in Supplementary Table 3.

### Chromatin immunoprecipitation

Cells were fixed with 1% formaldehyde, lysed with 0.5% NP-40, and centrifuged at 800×g to collect nuclei. The pellet was lysed with 1% SDS, sonicated, and incubated with IgG, anti-SLUG or anti-SNAIL antibody, and the immune complexes were pulled down using protein A/G beads (Santa Cruz Biotechnology). DNA in the precipitates was extracted by phenol‒chloroform-isoamyl alcohol (25:24:1), precipitated with ethanol, resolved in water, and subjected to PCR. The results are represented as the percentages of the input signal.

### Immunoblotting and immunoprecipitation

Proteins were subjected to SDS‒PAGE and transferred to Immobilon-P (Millipore). The membranes were incubated with primary and HRP-conjugated secondary antibodies in 5% skim milk and visualized using the ECL plus kit (Amersham Biosciences; Piscataway, NJ). For analysis of protein interactions, cell lysates were incubated with anti-MYC or anti-FLAG beads. The bound proteins were eluted using SDS or MYC/FLAG-tag peptides and loaded on SDS‒PAGE. All experiments were performed three or more times.

### Immunohistochemistry and immunofluorescence

For immunohistochemistry, tissue slides were deparaffinized, rehydrated, and heated to retrieve antigen. The slides were sequentially incubated with 3% H_2_O_2_, 2% horse serum, primary antibodies, and biotinylated secondary antibodies. The slides were treated with the DAB detection kit (Dako), counterstained with hematoxylin, and photographed at four high-power fields for each slide. Protein expression was analyzed using histoscore (the staining intensity (0–3) × the percentage of positive cells). Human gastric cancer tissue arrays were obtained from SuperBioChips (Seoul, Korea). Clinical information on the patients is summarized in Supplementary Table 4. For immunofluorescence, cells on a cover slide were fixed with 4% paraformaldehyde, permeabilized with 0.1% Triton-X-100 and 0.05% Tween-20, and sequentially incubated with primary antibodies and Alexa Fluor 488-conjugated secondary antibodies (Invitrogen). F-actin was stained with Alexa Fluor 488 phalloidin (Abcam). After incubation with DAPI (Sigma‒Aldrich), the slides were mounted in Faramount aqueous mounting medium (Dako). Fluorescence images were observed under a confocal microscope.

### Mass analysis to quantify intracellular fructose

Cell pellets were vortexed with an ice-cold extraction solvent (acetonitrile:methanol:water = 4:4:2, v/v/v) for 1 min, snap-frozen for 1 min, and centrifuged at 10,000 × *g* for 15 min. After being subjected to SpeedVac, the dried samples were dissolved in 50 µL of water and subjected to LC‒MS analysis. Sample separation was performed on a Hypersil Gold Amino column (150 × 2.1 mm, 1.9 μm, Thermo Scientific) using an Ultimate 3000 (Dionex; Idstein, Germany) with isocratic elution. The UPLC system was coupled to a Q Exactive Plus (Thermo Scientific) equipped with a heated electrospray ionization (HESI) source. The MS was operated in negative ion mode, and the scan range was from m/z 50 to 500 in targeted-sim mode.

### Measurement of fructose in the tumor tissues

On the 4th week after the intrasplenic implantation of MKN-28 cells, primary tumors in the spleens were excised and disrupted under liquid nitrogen. A piece (40 mg) of the frozen tumor was homogenized in ice-cold PBS and centrifuged at 18,000 × *g* for 15 min to obtain the supernatant. Fructose in the supernatant was measured using a fluorometric fructose assay kit (Sigma‒Aldrich).

### Simulation of molecular dynamics

Simulations of atomistic molecular dynamics were conducted on two KHK-A systems: fructose-free KHK-A and fructose-bound KHK-A. The initial structures of the two systems were obtained from PDB ID: 2hqq and 2hw1, respectively^27^. The model systems were solvated by TIP3P water molecules with 100 mM NaCl in the simulation box with a periodic boundary condition. The solvated system was then equilibrated at 310 K and 1 bar by performing NPT-ensemble MD simulations using the GROMACS program with the CHARMM36 force field. The modified Berendsen thermostat and Parrinello-Rahman barostat were used to maintain temperature (310 K) and pressure (1 bar). Full system periodic electrostatics were employed by the particle‒mesh Ewald method with a 1 Å grid spacing. The cutoff and switching distances for van der Waals forces were set to 12 Å and 10 Å, respectively. The bonds involving hydrogen were constrained to be rigid by using the LINCS algorithm. The MD system was equilibrated for 100 ns with a 2 fs time step, and the structure analysis was performed to simulate a further 200 ns run with recording every 25 ps.

### Bioinformatics analysis

Public data for mRNA levels in gastric cancer patients were obtained from GSE84437 (*n* = 433), GSE2685 (*n* = 30), and The Cancer Genome Atlas (TCGA) datasets. For survival analysis, 25 different types of cancer datasets from TCGA were used, and samples of each type of cancer were categorized into an AKR1B1 low-expression group and an AKR1B1 high-expression group by the median value. Kaplan‒Meier overall survival graphs were analyzed using GraphPad Prism software.

### Statistical analysis

All data were analyzed using Microsoft Excel 2013 or GraphPad Prism 5.0. The results are expressed as the means and standard deviation (SD) from three or more samples. The unpaired, two-sided Student’s *t* test or Mann‒Whitney *U* test was used for statistical analyses. Spearman correlation analysis was used to measure the correlation coefficient between AKR1B1 and CDH1 expression in the GSE2685 dataset. All statistical significances were considered when *P* values were less than 0.05.

## Results

### The polyol pathway is associated with gastric cancer progression

**Fig. 1:**
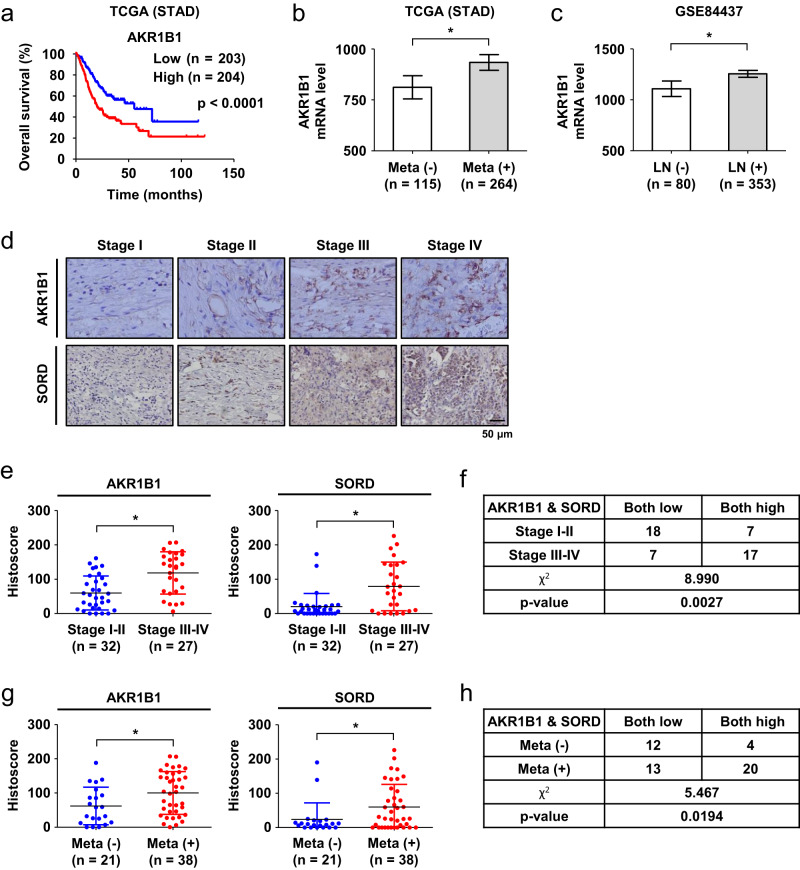
The polyol pathway is clinically associated with gastric cancer metastasis. **a** Kaplan–Meier analyses of overall survival in AKR1B1-low and AKR1B1-high gastric cancer patients. The *P* value was calculated by the log-rank test. **b** AKR1B1 mRNA expression in gastric cancer tissues of patients with and without metastasis. **c** AKR1B1 mRNA levels in gastric cancer tissues of patients with and without lymph node metastasis. **d** Representative images of human gastric cancer tissues immunostained with anti-AKR1B1 or anti-SORD antibodies. **e** AKR1B1 and SORD protein levels in low-stage (I and II) and high-stage (III and IV) tumors. **f** Chi-square analysis of AKR1B1/SORD expression and tumor stage. **g** AKR1B1 and SORD protein levels in nonmetastatic and metastatic tumors. **h** Chi-square analysis of AKR1B1/SORD expression and metastasis. * denotes *P* < 0.05 by the Mann‒Whitney *U* test.

To examine whether the polyol pathway is involved in gastric cancer progression, we focused on AKR1B1, which is the rate-limiting enzyme in the pathway. In gastric cancer patients, overall survival was lower in the AKR1B1-high group than in the AKR1B1-low group (Fig. 1a) but not in other cancer patients (Supplementary Fig. 1). The Cancer Genome Atlas (TCGA) revealed that AKR1B1 expression is higher in metastatic or in lymph node metastatic gastric cancers (Fig. 1b, c). Two enzymes, AKR1B1 and SORD, in the polyol pathway were analyzed in human gastric cancer tissues (Fig. 1d). When the specimens were divided into low (I and II) and high (III and IV) stage groups, AKR1B1 and SORD levels were elevated in the high stage group (Fig. 1e). A chi-square analysis revealed that gastric cancers with elevated expression of both AKR1B1 and SORD are more aggressive (Fig. 1f). Moreover, the protein levels were higher in the metastatic group (Fig. 1g), and the high expression of both proteins was associated with metastasis (Fig. 1h). Collectively, these clinical data suggest that the polyol pathway is associated with gastric cancer progression.

### High glucose enhances the metastatic potential in gastric cancer

**Fig. 2:**
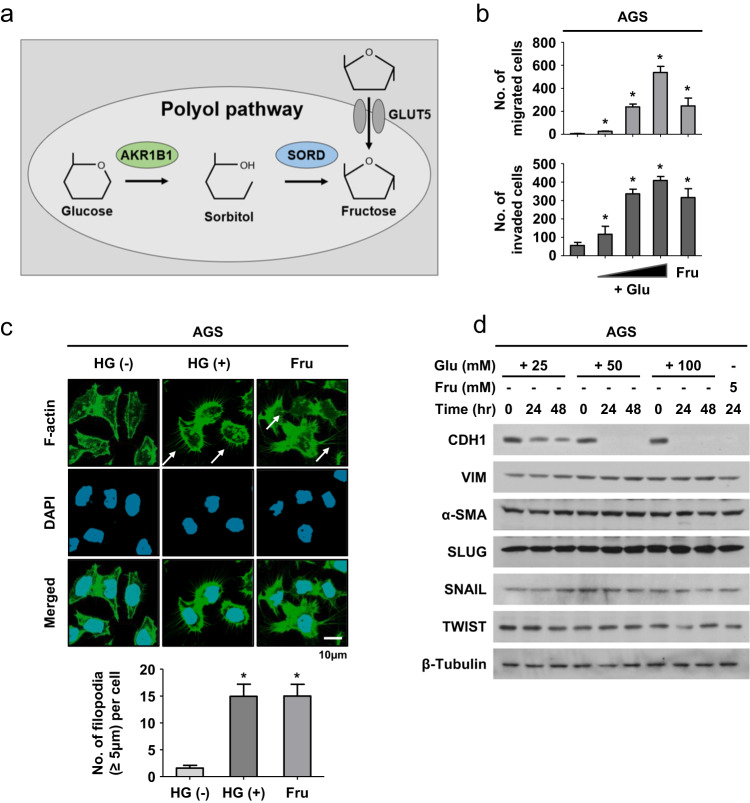
High glucose as well as fructose enhances metastatic potential in gastric cancer cells. **a** Graphical summary of the polyol pathway. **b** After AGS cells were incubated with glucose (+Glu; 25, 50, and 100 mM) or fructose (Fru, 5 mM) for 24 h, cell migration and invasion were analyzed in a Boyden chamber. The numbers of migrated or invaded cells are presented as bar graphs (means ± SDs, *n* = 3). * denotes *P* < 0.05 versus the euglycemic control. **c** F-actin (green) and nuclei (blue) were costained in AGS cells treated with high glucose (50 mM) or fructose (5 mM) for 24 h. White arrows indicate filopodia. The numbers of filopodia (≥5 μm) per cell are presented as bar graphs (mean ± SD, *n* = 3). *, *P* < 0.05 versus the control group. **d** AGS cells were treated with glucose or fructose for 24 or 48 h and subjected to Western blotting for EMT markers.

We hypothesized that high glucose facilitates cancer metastasis by increasing fructose via the polyol pathway (Fig. 2a). Based on the AKR1B1 and SORD levels (Supplementary Fig. 2a), AGS and MKN-45 cells were selected for experiments. High glucose and fructose both facilitated cell migration and invasion (Fig. 2b and Supplementary Fig. 2b, c). High glucose and fructose-induced F-actin rearrangement with filopodia-like extensions (Fig. 2c), indicating that the cells undergo EMT. Of the EMT markers, CDH1 (alternatively named E-cadherin) was markedly downregulated under high glucose and fructose conditions (Fig. 2d and Supplementary Fig. 2d). Thus, high glucose and fructose likely enhance the metastatic potential in gastric cancer cells by inducing EMT.

### The polyol pathway drives gastric cancer cell migration and invasion under high glucose

**Fig. 3:**
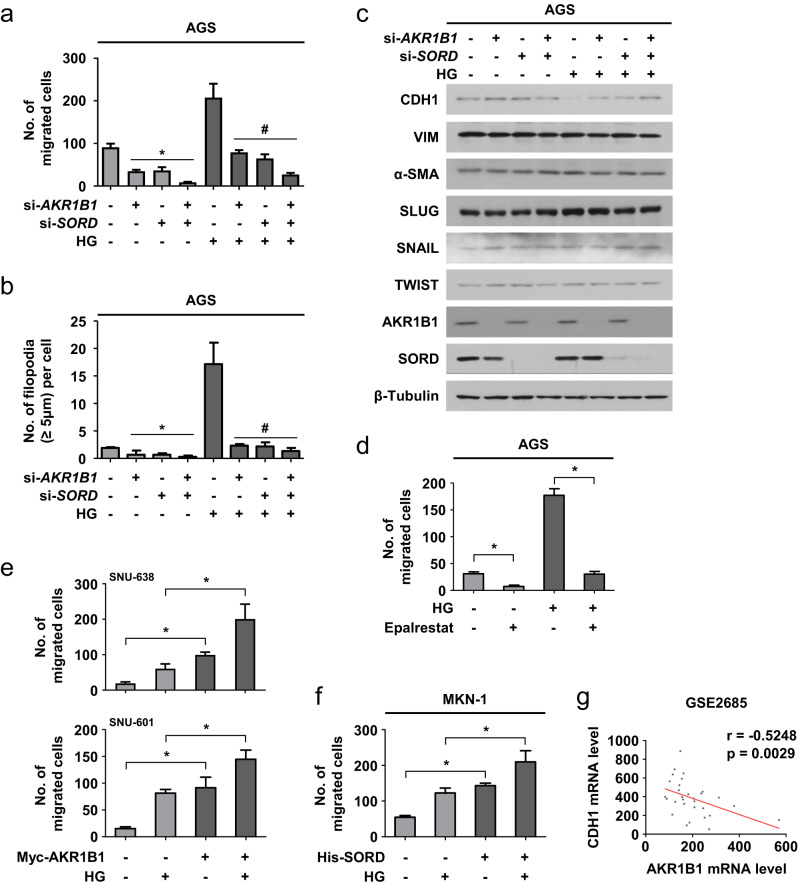
High glucose enhances the metastatic potential of gastric cancer through the polyol pathway. **a** AGS cells transfected with 80 nM siRNAs were treated with high glucose (HG) for 24 h and subjected to a migration assay. The numbers of migrated cells are presented as bar graphs (means ± SDs, *n* = 3). *, *P* < 0.05 versus the euglycemic control; #, *P* < 0.05 versus the hyperglycemic control. **b** In transfected AGS cells treated as indicated for 24 h, F-actin and nuclei were costained. Each bar represents the mean and SD (*n* = 3). **c** AGS cells were transfected with siRNAs and incubated with high glucose for 24 h. EMT protein markers were immunoblotted. **d** AGS cells were incubated with high glucose and 20 μM epalrestat for 24 h. The migrated cell numbers are presented as bar graphs (means ± SDs, *n* = 3). **e** SNU-638 and SNU-601 cells, which had been transfected with Myc-AKR1B1, were treated with high glucose for 24 h. The migrated cell numbers are presented as bar graphs (means + SDs, *n* = 3). **f** MKN-1 cells, which had been transfected with His(6)-SORD, were treated with high glucose for 24 h and subjected to a migration assay (means ± SDs, *n* = 3). **g** Spearman’s correlation analysis between AKR1B1 and CDH1 levels. The results were obtained from GSE2685 (*n* = 30). ‘*r*’ is the correlation coefficient. * and # denote *P* < 0.05 by Student’s *t* test.

Cell migration was attenuated by silencing AKR1B1 and/or SORD, and these effects were augmented under hyperglycemic conditions (Fig. 3a and Supplementary Fig. 3a, b). Cell numbers were reduced by ~10% after knockdown of both genes (Supplementary Fig. 3c), indicating that the attenuation of cell migration was not attributed to decreased cell number. Moreover, hyperglycemia-induced cytoskeletal rearrangement and CDH1 suppression were attenuated by AKR1B1 and/or SORD knockdown (Fig. 3b, c and Supplementary Fig. 3d, e). The AKR1B1 inhibitor epalrestat also attenuated cell migration (Fig. 3d and Supplementary Fig. 4a–d). In AKR1B1-deficient SNU-638 and SNU-601 cells, AKR1B1 expression increased cell migration and invasion under euglycemic conditions, which was augmented under hyperglycemic conditions (Fig. 3e and Supplementary Fig. 5a, b). In SORD-deficient MKN-1 cells, cell migration under hyperglycemic conditions was augmented by SORD overexpression (Fig. 3f and Supplementary Fig. 5c). Spearman correlation analysis using the GSE2685 gastric cancer dataset revealed that AKR1B1 expression was inversely correlated with CDH1 expression (Fig. 3g). Collectively, our results suggest that the polyol pathway drives the hyperglycemia-induced migration and invasion of gastric cancer cells.

### Polyol pathway-derived fructose mediates EMT and cell migration under hyperglycemic conditions

**Fig. 4:**
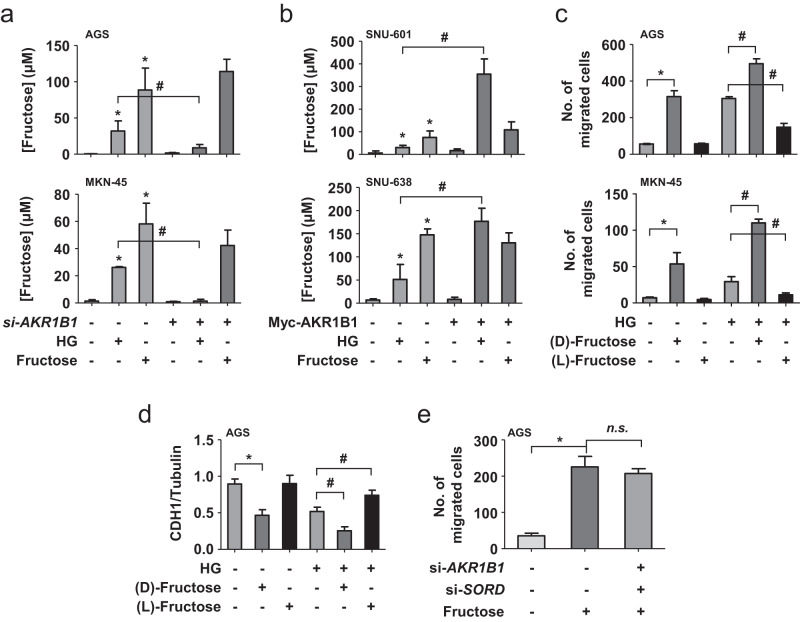
Polyol pathway-derived fructose mediates the increased metastatic potential under hyperglycemia. **a** AGS and MKN-45 cells were transfected with 80 nM si-AKR1B1. **b** SNU-601 and SNU-638 cells were transfected with MYC-AKR1B1 (1 μg). After stabilization for 24 h, the cells were incubated as indicated for 24 h. Cell extracts were subjected to MASS analyses to measure fructose levels. Fructose levels were normalized by packed cell volumes and are presented as bar graphs (means ± SDs, *n* = 3). **c** AGS and MKN-45 cells were treated with 5 mM D-fructose, 5 mM L-fructose, and/or 50 mM glucose for 24 h and subjected to a migration assay (means ± SDs, *n* = 3). **d** AGS cells, which were treated as indicated, were subjected to immunoblotting for EMT markers. The blots of CDH1 and β-tubulin were quantified using ImageJ (means ± SDs, *n* = 3). **e** Transfected AGS cells were treated with 5 mM fructose for 24 h and subjected to migration assays and immunoblotting. The migrated cell numbers (means ± SDs, *n* = 3) are presented as bar graphs. * and #, *P* < 0.05 (Student’s *t* test); n.s. not significant.

We tested whether fructose mediates hyperglycemia-induced migration and invasion. The intracellular level of fructose was enhanced by fructose supplementation and under hyperglycemic conditions, which was attenuated by silencing AKR1B1 in AGS and MKN-45 cells (Fig. 4a and Supplementary Fig. 6a, b). AKR1B1 expression increased the intracellular fructose levels in SNU-638 and SNU-601 cells (Fig. 4b and Supplementary Fig. 6a, c). In addition, L-fructose, which functionally competes with D-fructose, blocked hyperglycemia-induced cell migration (Fig. 4c and Supplementary Fig. 6d, e) and CDH1 suppression (Fig. 4d and Supplementary Fig. 6f). However, fructose-induced cell migration was not affected by AKR1B1 and SORD knockdown (Fig. 4e and Supplementary Fig. 6g). The polyol pathway under hyperglycemia is likely to stimulate cell migration by producing fructose rather than by regulating the fructose-mediated signaling pathway.

### High glucose triggers the fructose-dependent KHK-A signaling pathway

**Fig. 5:**
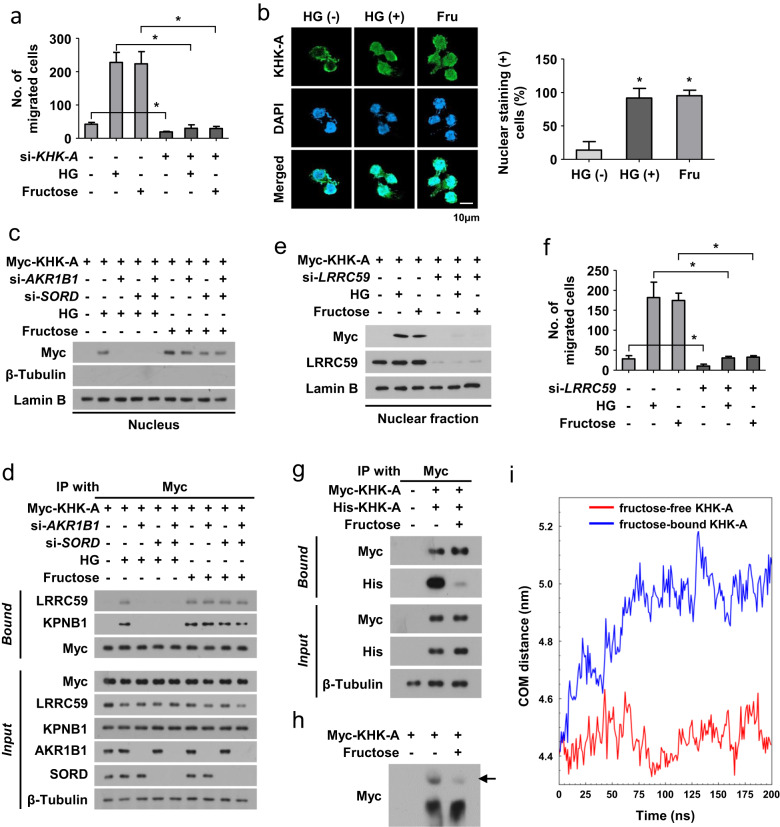
Polyol pathway-derived fructose activates the KHK-A signaling pathway. **a** Transfected AGS cells were incubated with high glucose for 24 h and subjected to a migration assay (means ± SDs, *n* = 3). * denotes *P* < 0.05. **b** AGS cells were incubated with high glucose or fructose for 24 h and immunostained (green for KHK-A and blue for DAPI). The percentage of nuclear KHK-A (+) cells was counted and presented as bar graphs (means ± SDs, *n* = 3). **c** After transfected AGS cells were incubated with high glucose or fructose for 24 h, cell lysates were fractionated into cytosolic and nuclear fractions. Myc-KHK-A was immunoblotted in the nuclear fraction. **d** Transfected AGS cells were treated with high glucose or fructose for 24 h and subjected to immunoprecipitation and immunoblotting. **e** Myc-tagged KHK-A was immunoblotted in the nuclear fraction from the transfected AGS cells incubated with high glucose or fructose for 24 h. **f** Transfected AGS cells were treated with high glucose or fructose for 24 h and subjected to a migration assay (means + SDs, *n* = 3). **g** AGS cells coexpressing Myc-KHK-A and His-KHK-A were incubated with fructose for 24 h and subjected to immunoprecipitation and immunoblotting. **h** The lysates of AGS cells expressing Myc-KHK-A were analyzed by native-PAGE followed by immunoblotting. The black arrow indicates the dimer form of KHK-A. **i** The center-of-mass (COM) distance variations between fructose-free and fructose-bound KHK-A throughout the MD simulation trajectory.

A recent study revealed the role of the ketohexokinase-A (KHK-A) signaling pathway in fructose-induced metastasis in breast cancer^28^. Exogenous fructose induces the nuclear translocation of KHK-A in association with KPNB1 and LRRC59, and in turn, KHK-A phosphorylates YWHAH, which recruits the repressor SLUG to the *CDH1* promoter. CDH1 suppression induces EMT and subsequently triggers breast cancer metastasis. Based on this scenario, we hypothesized that endogenous fructose synthesized via the polyol pathway stimulates the KHK-A signaling pathway. KHK exists as two isoforms, KHK-A and KHK-C. KHK-C converts fructose to fructose-1-phosphate, whereas KHK-A regulates cell signaling as a protein kinase. As KHK-C was not expressed in AGS and MKN-45 cells (Supplementary Fig. 7a), we focused on the KHK-A signaling pathway. Because ALDOB, ALOX12, and KHK-A have been reported to promote fructose-induced cancer metastasis^29,30^ (Supplementary Fig. 7b), we determined which of the three participated in hyperglycemia-induced cell migration. Cell migration was attenuated by KHK-A knockdown but not by ALDOB or ALOX12 knockdown. When KHK-A was silenced, hyperglycemia and fructose showed marginal effects on cell migration (Fig. 5a and Supplementary Fig. 7c–e). Immunofluorescence and immunoblotting analyses revealed that hyperglycemia induced the nuclear translocation of endogenous and expressed KHK-A but did not induce the nuclear translocation of expressed KHK-C (Fig. 5b and Supplementary Fig. 8a–c). This effect of hyperglycemia was attenuated by silencing AKR1B1 and/or SORD (Fig. 5c and Supplementary Fig. 8d, e). We also found that KHK-A interacted with LRRC59 and KPNB1 under hyperglycemic conditions, which was inhibited by AKR1B1 and/or SORD knockdown (Fig. 5d). LRRC59 was essential for the hyperglycemia-induced nuclear translocation of KHK-A and cell migration (Fig. 5e, f and Supplementary Fig. 9a). We next examined how fructose facilitates KHK-A binding to LRRC59 and KPNB1. Given that KHK-A exists as a dimer^27^, we tested whether fructose dissociates the KHK-A dimer to allow the KHK-A monomer to associate with other proteins. To identify the dimerization of KHK-A, we coexpressed Myc- and His(6)-tagged KHK-A in AGS and MKN-45 cells and performed coimmunoprecipitation. Myc-KHK-A and His-KHK-A were found to associate with each other. Surprisingly, the dimerization was weakened by fructose (Fig. 5g and Supplementary Fig. 9b). On native PAGE, the dimer of Myc-KHK-A was shown as an upper band, which faded away under fructose supplementation (Fig. 5h). The fructose-induced dissociation of the KHK-A dimer is further supported by the crystallography-determined structures of fructose-free and fructose-bound KHK-A (Supplementary Fig. 10a)^27^. The superimposed structures reveal a distinct difference in the side chain orientations of Tyr112 and Tyr113 in the interfacial region between the two monomers, while other residues retain the same orientations (Supplementary Fig. 10b). The observed shift of the Tyr phenol rings toward the same monomer region in the fructose-bound state suggests a loss of intermonomer interaction. To further understand the molecular basis of this orientation shift of the Tyr side chains upon fructose binding, we performed atomistic molecular dynamics (MD) simulations. In the MD trajectory analysis, the center-of-mass (COM) distance between two monomers is increased by fructose binding (Fig. 5i), suggesting dissociation of the dimer. The binding sites of fructose on KHK-A comprise Asp15, Gly41, Asn45, and Asp258. In the unbound state, fructose-free Asp15 is more inclined to form hydrogen bonds with the backbone oxygen of Ala97 and Val13, disturbing their hydrophobic interaction with Tyr112 (Supplementary Fig. 11a, c). In contrast, upon fructose binding to Asp15, it disrupts the hydrogen bonds between Asp15 and both Ala97 and Val13, enhancing the propensity of Ala97 and Val13 to interact with Tyr112, which shifts the Tyr112 phenol ring orientation away from the monomer/monomer interface (Supplementary Fig. 11b, d). This shift in Tyr112, altering its interaction states in the monomer/monomer interface, is accompanied by a similar reorientation in Tyr13, another key contributor to monomer/monomer interactions. These shifts collectively result in the weakening of KHK-A dimerization.

### Polyol pathway-derived fructose induces EMT through the KHK-A signaling pathway

**Fig. 6:**
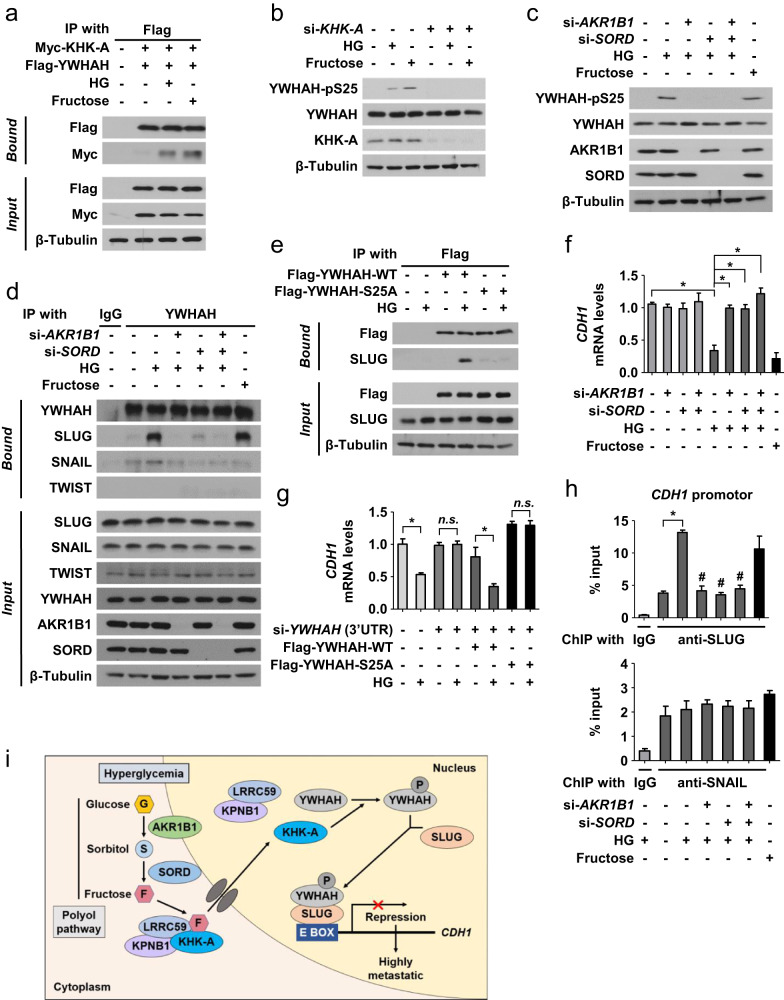
Polyol pathway-derived fructose induces EMT through the KHK-A signaling pathway. **a** AGS cells expressing Myc-KHK-A and Flag-YWHAH were incubated as indicated, and the cell lysates were immunoprecipitated and immunoblotted. **b** In AGS cells, KHK-A-dependent phosphorylation of YWHAH at Ser25 was evaluated by immunoblotting. **c** Transfected AGS cells were treated with high glucose or fructose for 24 h and subjected to immunoblotting. **d** AGS cells were subjected to immunoprecipitation and immunoblotting. **e** The lysates of AGS cells expressing Flag-YWHAH (or S25A) were immunoprecipitated and immunoblotted. **f** In AGS cells, the mRNA levels of CDH1 were analyzed by RT‒qPCR (means ± SDs, *n* = 3). **g** The mRNA levels (the mean ± SD, *n* = 3) of CDH1 were measured by RT‒qPCR in AGS cells. *, *P* < 0.05; n.s. not significant. **h** The transfected AGS cells were subjected to ChIP‒qPCR using anti-SLUG or anti-SNAIL antibodies. *, *P* < 0.05 versus the euglycemic si-control; #, *P* < 0.05 versus the hyperglycemic si-control. **i** The proposed mechanism underlying diabetes-induced cancer metastasis.

Under high glucose and fructose conditions, KHK-A interacted with YWHAH (Fig. 6a) and phosphorylated YWHAH at S25 (Fig. 6b). More importantly, hyperglycemia-induced phosphorylation of YWHAH was almost completely attenuated by silencing AKR1B1 and/or SORD (Fig. 6c), strongly suggesting that polyol pathway-derived fructose drives S25 phosphorylation of YWHAH. Of the three *CDH1* repressors SNAIL, SLUG, and TWIST, YWHAH robustly interacted with SLUG in gastric cancer cells, which was inhibited by silencing AKR1B1 and/or SORD (Fig. 6d and Supplementary Fig. 12a). Under fructose supplementation, the KHK-A-dependent phosphorylation of YWHAH and the interaction of YWHAH with SLUG were not affected by AKR1B1 and SORD knockdown (Supplementary Fig. 12b, c), indicating that fructose acts as the final effector of the polyol pathway. Given that the YWHAH_S25A mutant failed to interact with SLUG, the KHK-A-dependent S25 phosphorylation of YWHAH might be critical for the interactions under hyperglycemic conditions (Fig. 6e). Next, we found that the polyol pathway is responsible for *CDH1* repression under hyperglycemic conditions (Fig. 6f and Supplementary Fig. 13a). As expected, AKR1B1 and SORD knockdown did not affect *CDH1* repression under fructose supplementation (Supplementary Fig. 13b, c). Hyperglycemia-induced *CDH1* repression was rescued by YWHAH knockdown, and wild-type YWHAH, not the S25A mutant, repressed *CDH1* (Fig. 6g). The recruitment of SLUG to the *CDH1* promoter was augmented by hyperglycemia, which was attenuated by silencing AKR1B1 and/or SORD (Fig. 6h and Supplementary Fig. 13d). In contrast, SNAIL binding to the *CDH1* promoter was not regulated by the polyol pathway. We also found that S25 phosphorylation of YWHAH is critical for SLUG, but not SNAIL, recruitment to the *CDH1* promoter in a glucose-dependent manner (Supplementary Fig. 14a). Moreover, S25 phosphorylation of YWHAH was critical for cell invasion under hyperglycemic conditions (Supplementary Fig. 14b). Taken together, these results suggest that polyol pathway-derived fructose enhances the metastatic potential of gastric cancer cells via the KHK-A-YWHAH-SLUG pathway. The polyol pathway and KHK-A signaling for gastric cancer metastasis are summarized in Fig. 6i.

### Diabetes-induced metastasis of gastric cancer in a spleen-to-liver metastasis model

**Fig. 7:**
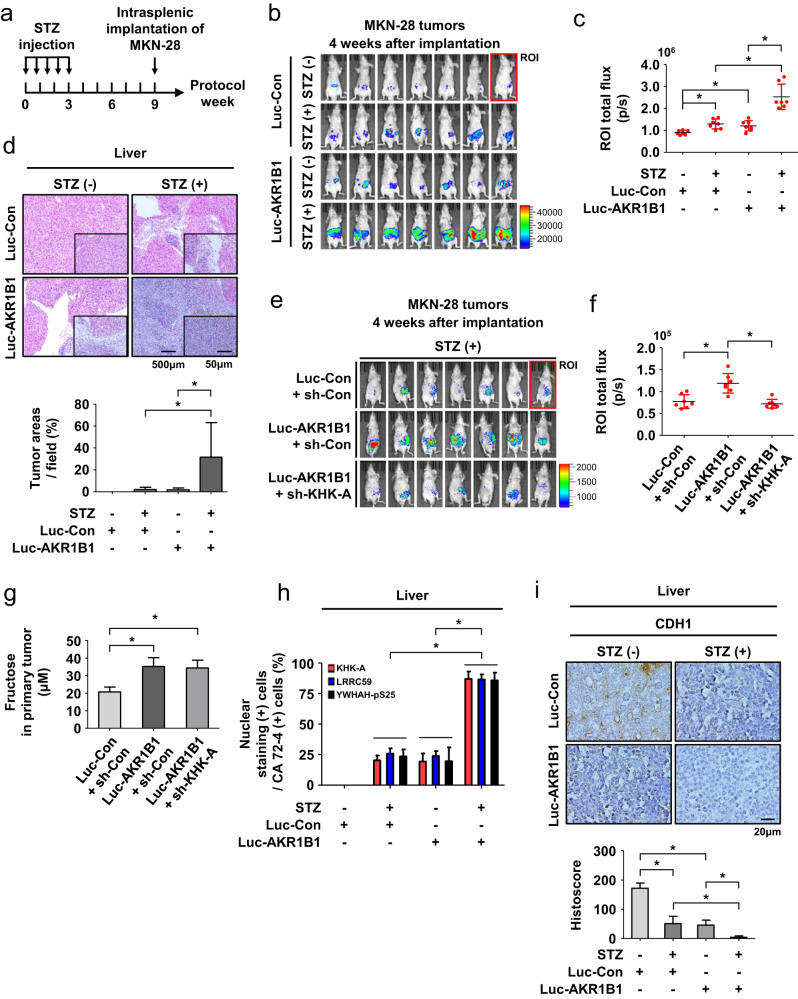
Hyperglycemia facilitates the spleen-to-liver metastasis of gastric cancer grafts in mice. **a** Schematic diagram of an animal model for hyperglycemia-induced metastasis of gastric cancer. STZ was intravenously injected to induce diabetes. MKN-28 stable cell lines were slowly implanted in the spleen using a syringe. **b** Four weeks after the intrasplenic implantation of tumor cells, bioluminescence images of tumor-bearing mice were monitored using Xenogen IVIS spectroscopy. The color bar represents tumor intensity from purple (low) to red (high). **c** Quantitative analysis of bioluminescence emission in total flux (photons/s/cm^2^/sr) measured 4 weeks after tumor implantation. **d** On the 4th week after tumor implantation, livers were excised from mice. Representative images of H&E-stained livers (top panel) and tumor areas per field were quantified using ImageJ (bottom panel). **e** MKN-28 stable cell lines expressing the indicated genes were grafted into the spleens of diabetic mice. Bioluminescence images of the mice were taken. **f** Bioluminescence intensities of tumor-bearing mice were quantified. **g** Fructose levels were measured in primary tumors excised from spleens. **h** The excised livers were costained with the indicated antibodies and anti-CA72-4 antibody. All tissues were stained with fluorescent dyes for visualization. The percentage of nuclear KHK-A, LRRC59, or YWHAH-pS25 (+) cells in CA 72-4 (+) cells was counted and presented as bar graphs. **i** Representative photographs of liver tissues immunostained with anti-CDH1 antibody (top panel). The expression levels were analyzed based on histoscore (bottom panel). Each result in the graph is presented as the mean and SD. * denotes *P* < 0.05 by Student’s *t* test.

We next investigated whether the polyol pathway promotes gastric cancer metastasis in mice with diabetes. We used a model of spleen-to-liver metastasis because gastric cancer frequently metastasizes to the liver. The experimental schedule is shown in Fig. 7a. We first established stable MKN-28 cell lines, which were originally derived from intestinal gastric cancer, that stably coexpressed luciferase and AKR1B1 and verified the luciferase activity and AKR1B1 expression (Supplementary Fig. 15a, b). In the streptozotocin (STZ)-treated mice in groups “b” and “d”, blood glucose levels were substantially increased to approximately 600 mg/dL (Supplementary Fig. 15c). Significant weight loss was observed in group “d” (Supplementary Fig. 15d). By monitoring bioluminescence emission from cancer cells, we observed more severe liver metastasis in the diabetic controls than in the nondiabetic controls. AKR1B1 overexpression promoted metastasis in nondiabetic mice and, to a greater extent, in diabetic mice (Fig. 7b, c). Histological examination of liver tissues verified that bioluminescence was emitted from metastasizing cancer cells (Fig. 7d). Representative images of liver metastases are shown in Supplementary Fig. 15e, and metastasizing cancer cells within tumors were identified by immunostaining for CA 72-4 (Supplementary Fig. 15f). We next evaluated whether AKR1B1-driven metastasis is mediated by the KHK-A signaling pathway. Cell lines were established to stably express AKR1B1/sh-*KHK-A* (or sh-control) with luciferase. Luciferase activity, AKR1B1 expression, and KHK-A knockdown were assessed in the cell lines (Supplementary Fig. 16a–d). The blood glucose level was maintained at approximately 600 mg/dL in the STZ-treated mice, and significant body weight loss was found in group “f” but not in group “g” (Supplementary Fig. 16e, f). On the 4th week after tumor implantation, bioluminescence analyses showed that the tumor metastasis induced by AKR1B1 overexpression was strongly attenuated by KHK-A knockdown (Fig. 7e, f). We next examined whether the polyol pathway promotes fructose generation in the primary tumors of diabetic mice and found that the fructose levels were significantly higher in the AKR1B1-expressing tumors than in the control tumors (Fig. 7g and Supplementary Fig. 16g). Then, we examined whether diabetes-induced metastasis is mediated by the KHK-A signaling pathway. Nuclear localization of KHK-A/LRRC59 and S25 phosphorylation of YWHAH were significantly increased in the AKR1B1-expressing tumors of diabetic mice (Fig. 7h and Supplementary Fig. 17a–c). In addition, CDH1 was downregulated in the liver metastases of diabetic mice, and this effect was further augmented in the AKR1B1-expressing tumors (Fig. 7i).

### Diabetes-induced metastasis of gastric cancer in a subcutaneous xenograft model

**Fig. 8:**
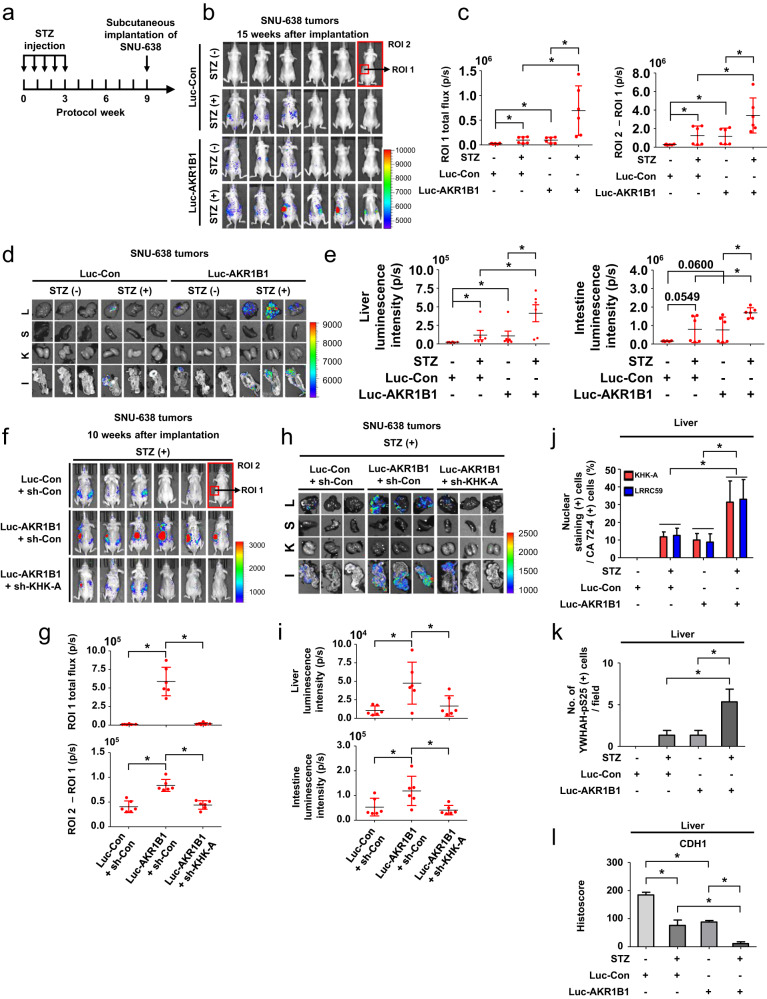
Hyperglycemia facilitates the distant metastasis of gastric cancer grafts in mice. **a** Schematic diagram of the gastric cancer xenograft study. SNU-638 stable cell lines were injected subcutaneously into the left flank of mice. **b** On the 15th week after tumor inoculation, bioluminescence images of the mice were taken. **c** Bioluminescence intensities of grafted tumors (ROI 1) and metastases (ROI 2 – ROI 1) were quantitatively analyzed. **d** Bioluminescence images in excised organs were captured (L = liver, S = spleen, K = kidney, I = intestine). The color bar represents tumor intensity from purple (low) to red (high). **e** Quantitative analysis of bioluminescence emission in the livers and intestines. **f** On the 10th week after the subcutaneous implantation of tumor cells, bioluminescence images of mice were taken. **g** Quantitative analysis of bioluminescence emission of grafted tumors (ROI 1) and metastases (ROI 2 – ROI 1) measured 10 weeks after tumor implantation. **h** Bioluminescence images of organs were captured. **i** Bioluminescence intensities in the livers and intestines were analyzed. **j** The excised livers were stained with antibodies against KHK-A or LRRC59 and costained with anti-CA 72-4 antibody. For visualization of protein expression, fluorescent dyes were used. The percentage of nuclear KHK-A or LRRC59 expression in CA72-4 (+) cells was quantified and is presented as bar graphs. **k** Livers were subjected to immunofluorescence staining. All tissues were stained with an antibody against YWHAH-pS25 and DAPI. **l** The liver sections were immunostained with anti-CDH1, which was evaluated using histoscore. Each result in the graph is presented as the mean and SD. * denotes *P* < 0.05 by Student’s *t* test.

For a subcutaneous xenograft model, SNU-638, which originally arose from a diffuse type of gastric cancer, was established to stably coexpress luciferase and AKR1B1 (Supplementary Fig. 18a, b). Mice (6 per group) were randomly allocated and subjected to tumor graft (Fig. 8a). Blood glucose levels verified that diabetes was stably maintained after tumor implantation (Supplementary Fig. 18c). In the diabetic group, the body weights of the mice declined in the late period of the experiment (Supplementary Fig. 18d). On the 15th week after tumor injection, bioluminescence imaging analyses revealed tumor growth at the injection sites and abdominal metastases in the AKR1B1-expressing tumors, and this effect was further enhanced in diabetic mice (Fig. 8b). The luminescence values of the region of interest (ROI) were used for the quantitative analysis of primary tumor growth and local tumor invasion (ROI 1) or distant metastasis (ROI 2–ROI 1) (Fig. 8c). Bioluminescence emissions of the excised livers and intestines were robustly detected in group “d” (Fig. 8d, e and Supplementary Fig. 18e), with weak emissions in groups “b” and “c” and little emission in group “a”. CA72-4 marked metastasizing cancer cells (Supplementary. Fig. 18f, g). We also established stable SNU-638 cell lines expressing luciferase and/or AKR1B1 and/or sh-*KHK-A* and implanted them into diabetic mice (Supplementary Fig. 19a–d). Blood glucose levels and body weights were monitored once a week (Supplementary Fig. 19e, f). When KHK-A was silenced, AKR1B1 overexpression failed to promote local tumor expansion and tumor metastasis in diabetic mice (Fig. 8f, g). We assessed bioluminescence emissions of the excised livers and intestines and found that AKR1B1-induced tumor metastasis was almost completely attenuated by KHK knockdown (Fig. 8h, i and Supplementary Fig. 19g). Moreover, the nuclear localization of KHK-A/LRRC59, S25 phosphorylation of YWHAH, and suppression of CDH1 expression were significantly enhanced in the AKR1B1-expressing tumors of diabetic mice (Fig. 8j–l and Supplementary Fig. 20a–d). Collectively, these results suggest that diabetes promotes gastric cancer metastasis through the polyol and KHK-A signaling pathways.

## Discussion

Most studies investigating the role of diabetes in cancer progression have focused on the signaling pathways and gene expression altered by high glucose^16–18^. Here, we explored the mechanism by investigating the role of fructose rather than glucose per se. Under hyperglycemia, gastric cancer cells acquired an increased potential for migration and invasion with EMT, all of which occurred depending on fructose synthesis via the polyol pathway. Under hyperglycemia, polyol pathway-derived fructose was sufficient to stimulate the KHK-A signaling pathway. In two different animal models mimicking gastric cancer metastasis, we found that metastasis was significantly increased in diabetic mice bearing AKR1B1-overexpressing tumors. Mechanistically, polyol pathway-derived fructose triggered the nuclear translocation of KHK-A, which phosphorylated YWHAH and repressed the *CDH1* gene by recruiting SLUG to its promoter, thereby inducing EMT. Collectively, we propose that the connection between the polyol pathway and KHK-A signaling pathway plays a crucial role in diabetes-induced gastric cancer metastasis. Based on this mechanism, we also suggest that the enzymes AKRIB1 and KHK-A could be potential targets for lowering metastatic risk in patients with gastric cancer.

Gastric cancer is the fifth most common cancer and the third most common cause of cancer-related deaths worldwide^2^. Several clinical studies have recently reported a significant correlation between diabetes and gastric cancer progression^11,13^. In addition, a meta-analysis revealed that hyperglycemia correlates with gastric cancer risk (HR, 1.11; 95% CI, 0.98–1.26)^31^. The 5-year survival rate of gastric cancer patients with diabetes is significantly lower than that of nondiabetic patients^32^. Although gastric cancer is highly prevalent, the clinical outcomes of patients without metastasis are favorable^33^. Indeed, gastrectomy is the best way to eradicate gastric cancers, and patients without a stomach can maintain relatively healthy lives with nutritional supplements. Thus, it is important to prevent metastasis in patients with gastric cancer. Our results provide a theoretical basis for strict control of hyperglycemia in cancer patients with diabetes to prevent metastasis.

According to Lauren’s criteria, gastric cancer can be classified into intestinal and diffuse types^34^. The intestinal type forms localized masses removable surgically, but the diffuse type infiltrates into the surrounding tissues and shows a worse prognosis^34^. MKN-28 and SNU-638 were used as representative cells for intestinal and diffuse types, respectively. Considering the distinct properties of these cells, we also adopted two xenograft models: intrasplenic implantation of MKN-28 cells and subcutaneous implantation of SNU-638 cells. Interestingly, MKN-28 and SNU-638 metastasized to the liver in different histological patterns. Within mouse livers, MKN-28 cells formed large nodules, whereas SNU-638 cells infiltrated into the liver parenchyma without clumping. Even in tumor xenografts, gastric cancer cells may retain growth properties inherited from their origins.

Several studies have reported the effect of hyperglycemia on gene expression. Hyperglycemia induces MMP2 expression in cholangiocarcinoma by activating STAT3^35^, upregulates MMP9 in lung cancer by inducing HMOX1^36^, and upregulates MMP2/9 in breast cancer^37^. The upregulation of MMPs could be responsible for hyperglycemia-induced cancer metastasis^38^. In lung cancer cells, hyperglycemia induces TGF-β secretion, which stimulates EMT and cell migration^39^. Hyperglycemia also triggers the degradation of the p53 activator HIPK2^40^, inhibiting p53-dependent apoptosis^41^. HIF1A, which expresses many hypoxia-induced genes, is also upregulated by hyperglycemia and consequently induces VEGF and HMOX1, thereby promoting angiogenesis and tumor growth^42^. However, to the best of our knowledge, little is known about the signaling pathways that initiate these hyperglycemic effects. Moreover, it remains unclear how cancer cells sense glucose levels. Here, we suggest that polyol pathway-derived fructose stimulates cancer metastasis and that KHK-A appears to act as a fructose sensor.

ALDOB and ALOX12 have been reported to be involved in fructose-induced cancer metastasis^28–30^. KHK converts fructose to fructose-1-phosphate, and ALDOB converts fructose-1-phosphate to glyceraldehyde and dihydroxyacetone phosphate. Colorectal cancer cells undergo metabolic reprogramming after liver metastasis. ALDOB is transcriptionally induced by GATA6 and promotes fructose metabolism, which provides metastasizing cancer cells with the energy and materials necessary for increased growth^29^. The lipoxygenase ALOX12 produces 12-HETE, which induces inflammation and promotes cancer progression. In breast cancer, fructose upregulates ALOX12 and a corresponding increase in 12-HETE, thereby promoting lung metastasis^30^. In contrast, KHK-A acts as a nuclear protein kinase upon fructose stimulation and represses *CDH1*, thereby facilitating breast cancer metastasis^28^. However, our results showed that fructose-induced cell migration and invasion were not attenuated by silencing either ALDOB or ALOX12, indicating that KHK-A primarily contributed to the prometastatic effect of fructose.

In general, the downregulation of CDH1 and the upregulation of CDH2 (N-cadherin) and VIM (vimentin) are regarded as hallmarks of EMT. However, in EMT under hyperglycemia (this study) or fructose supplementation^28^, CDH1 was dramatically repressed, but CDH2 and VIM were not induced. Then, is it possible that EMT is induced by only CDH1 repression irrespective of CDH2 and VIM? Notably, EMT does not always require all the hallmarks for EMT. Indeed, many clinical studies have revealed that the loss of CDH1 is an independent marker for gastric cancer progression^43^. Furthermore, in cell experiments, EMT has been reported to be induced by genetically repressing only CDH1^44^. Therefore, it is not surprising that the remarkable suppression of only CDH1 underlies the EMT induction by hyperglycemia.

In addition to the polyol pathway, AKR1B1 participates in various signaling pathways by metabolizing lipid aldehyde and prostaglandins. For instance, AKR1B1 stimulates PLC and PKC by reducing GSH-aldehyde under increased oxidative stress, thereby activating the NF-κB pathway^45,46^. NF-κB plays crucial roles in tumor progression by transcriptionally inducing various cytokines in the tumor microenvironment. AKR1B1 is also known to regulate prostaglandin metabolism. This molecule functions to convert prostaglandin H2 (PGH2) to prostaglandin F2α (PGF2A), which promotes tumor growth by activating the PKC-MAPK, PKA-GSK3B, and PI3K-mTOR pathways^47,48^. AKR1B1 may promote tumor metastasis via nonpolyol pathways such as NF-κB and PGF2A. At least in our experimental settings, however, the polyol pathway seems to be the main mechanism underlying hyperglycemia-induced metastasis in gastric cancer. This argument could be strongly supported by our results showing that the EMT of cancer cells and the metastasis of grafted tumors were substantially attenuated by KHK-A inhibition because the nonpolyol pathways are independent of KHK-A signaling.

Several AKR1B1 inhibitors are in clinical trials as therapeutic drugs for diabetic complications^49,50^. According to our results, AKR1B1 inhibitors could be potential drugs for preventing cancer metastasis in diabetic patients. If needed, AKR1B1 inhibitors can be coadministered with conventional anticancer drugs in cancer patients with diabetes. In some cases, AKR1B1 inhibitors could be used for dual purposes to inhibit diabetic complications or cancer exacerbation. However, KHK-A inhibitors could also have potential therapeutic benefits and may prevent gastric cancer metastasis in diabetic patients. The KHK inhibitor PF-06835919, which is currently under clinical trials as a therapeutic agent for nonalcoholic steatosis and steatohepatitis^51^, is an emerging agent for the prevention of cancer metastasis. Theoretically, it is plausible to test a combination therapy using AKR1B1 and KHK inhibitors to lower the risk of cancer metastasis in patients with diabetes.

Despite the high homology, KHK-A and KHK-C have different biochemical functions^52,53^. Several studies have investigated KHK-C-driven fructose flux as an underlying mechanism of fructose-induced cancer progression. High fructose levels have been reported to provide fuel and building blocks necessary for cancer growth and metastasis^29,54^. However, notably, KHK-A is predominantly expressed in most cancer cells, whereas KHK-C is rarely expressed. Since KHK-A has poor fructose phosphorylation activity, it is expected that fructose metabolism does not profoundly contribute to the progression of most cancers lacking KHK-C. However, the endogenous role of KHK-A remains to be uncovered^28^. A recent study identified the function of KHK-A as a protein kinase. KHK-A enhances nucleic acid synthesis by phosphorylating and activating PRPS1, augmenting cell proliferation^55^. KHK-A also phosphorylates YWHAH and consequently suppresses CDH1 expression, thereby promoting EMT and metastasis^28^. Based on these reports, KHK-A is likely to act as a protein kinase to facilitate cancer growth and metastasis.

In conclusion, we report that the excessive production of fructose via the polyol pathway and the fructose-triggered KHK-A signaling pathway drives gastric cancer metastasis under hyperglycemic conditions. For patients with comorbid gastric cancer and diabetes, we strongly recommend strict control of blood glucose levels to prevent diabetes-induced cancer exacerbation. We also propose that the polyol and KHK-A signaling pathways could be potential targets to prevent and treat cancer metastasis in patients with diabetes.

### Supplementary information

Supplementary Information
