# Supplementary material for: The polyol pathway and nuclear ketohexokinase A signaling drive hyperglycemia-induced metastasis of gastric cancer
Source: Exp Mol Med. 2024 Jan 10;56(1):220–34. doi: 10.1038/s12276-023-01153-3 (PMC10834943; doi:10.1038/s12276-023-01153-3)
Supplement: Supplementary file 1 — Supplementary Information [file 12276_2023_1153_MOESM1_ESM.pdf]

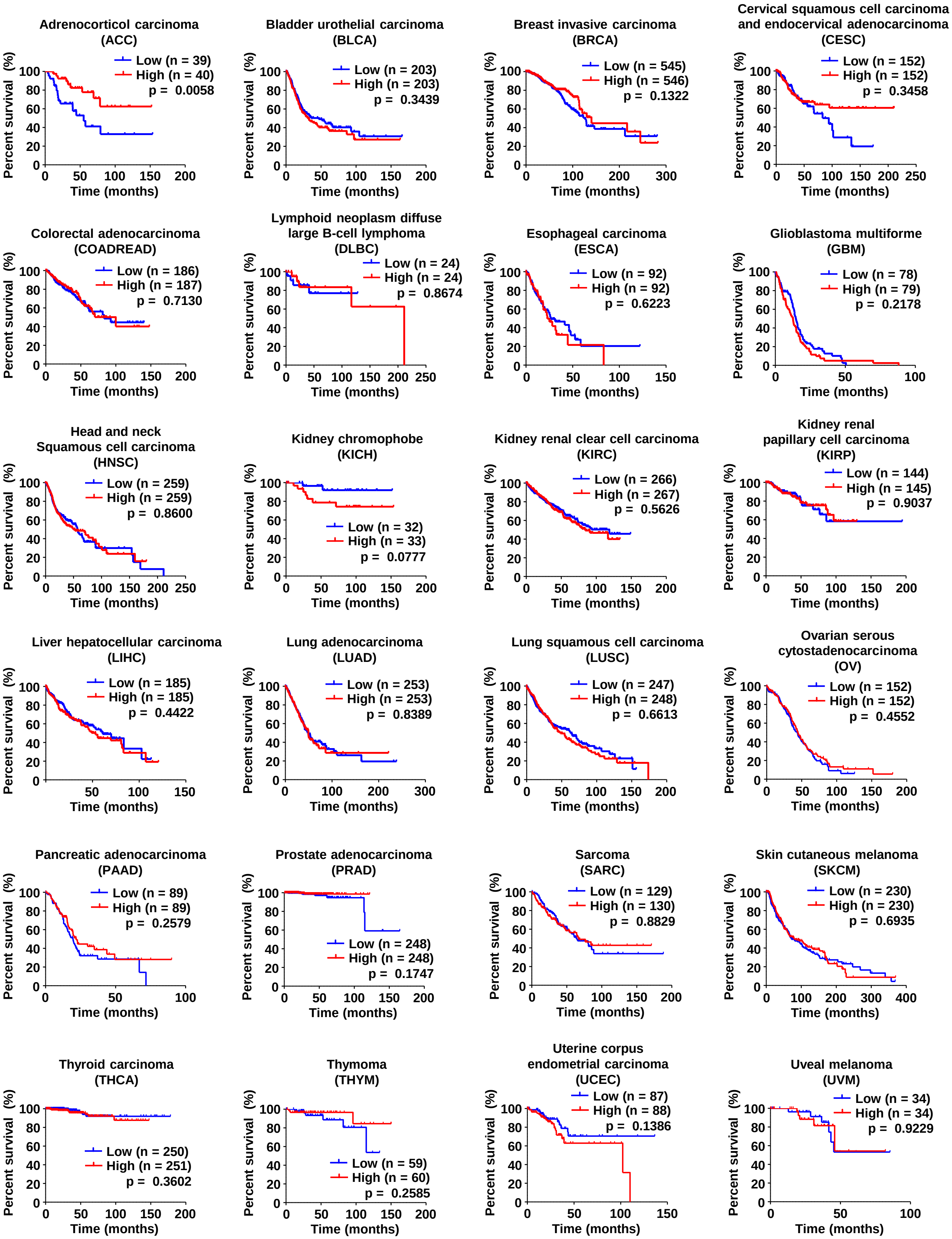
Adrenocorticol carcinoma (ACC)
Low (n = 39)
High (n = 40)
p = 0.0058
Bladder urothelial carcinoma (BLCA)
Low (n = 203)
High (n = 203)
p = 0.3439
Breast invasive carcinoma (BRCA)
Low (n = 545)
High (n = 546)
p = 0.1322
Cervical squamous cell carcinoma and endocervical adenocarcinoma (CESC)
Low (n = 152)
High (n = 152)
p = 0.3458
Colorectal adenocarcinoma (COADREAD)
Low (n = 186)
High (n = 187)
p = 0.7130
Lymphoid neoplasm diffuse large B-cell lymphoma (DLBC)
Low (n = 24)
High (n = 24)
p = 0.8674
Esophageal carcinoma (ESCA)
Low (n = 92)
High (n = 92)
p = 0.6223
Glioblastoma multiforme (GBM)
Low (n = 78)
High (n = 79)
p = 0.2178
Head and neck Squamous cell carcinoma (HNSC)
Low (n = 259)
High (n = 259)
p = 0.8600
Kidney chromophobe (KICH)
Low (n = 32)
High (n = 33)
p = 0.0777
Kidney renal clear cell carcinoma (KIRC)
Low (n = 266)
High (n = 267)
p = 0.5626
Kidney renal papillary cell carcinoma (KIRP)
Low (n = 144)
High (n = 145)
p = 0.9037
Liver hepatocellular carcinoma (LIHC)
Low (n = 185)
High (n = 185)
p = 0.4422
Lung adenocarcinoma (LUAD)
Low (n = 253)
High (n = 253)
p = 0.8389
Lung squamous cell carcinoma (LUSC)
Low (n = 247)
High (n = 248)
p = 0.6613
Ovarian serous cytostadenocarcinoma (OV)
Low (n = 152)
High (n = 152)
p = 0.4552
Pancreatic adenocarcinoma (PAAD)
Low (n = 89)
High (n = 89)
p = 0.2579
Prostate adenocarcinoma (PRAD)
Low (n = 248)
High (n = 248)
p = 0.1747
Sarcoma (SARC)
Low (n = 129)
High (n = 130)
p = 0.8829
Skin cutaneous melanoma (SKCM)
Low (n = 230)
High (n = 230)
p = 0.6935
Thyroid carcinoma (THCA)
Low (n = 250)
High (n = 251)
p = 0.3602
Thymoma (THYM)
Low (n = 59)
High (n = 60)
p = 0.2585
Uterine corpus endometrial carcinoma (UCEC)
Low (n = 87)
High (n = 88)
p = 0.1386
Uveal melanoma (UVM)
Low (n = 34)
High (n = 34)
p = 0.9229
Percent survival (%)
Time (months)

Supplementary Figure 1.

Kaplan–Meier overall survival analysis in various cancers on TCGA datasets. Based on the median value, the tumor cohorts are divided into two groups, AKR1B1-low and AKR1B1-high. Significance is calculated by log-rank test.

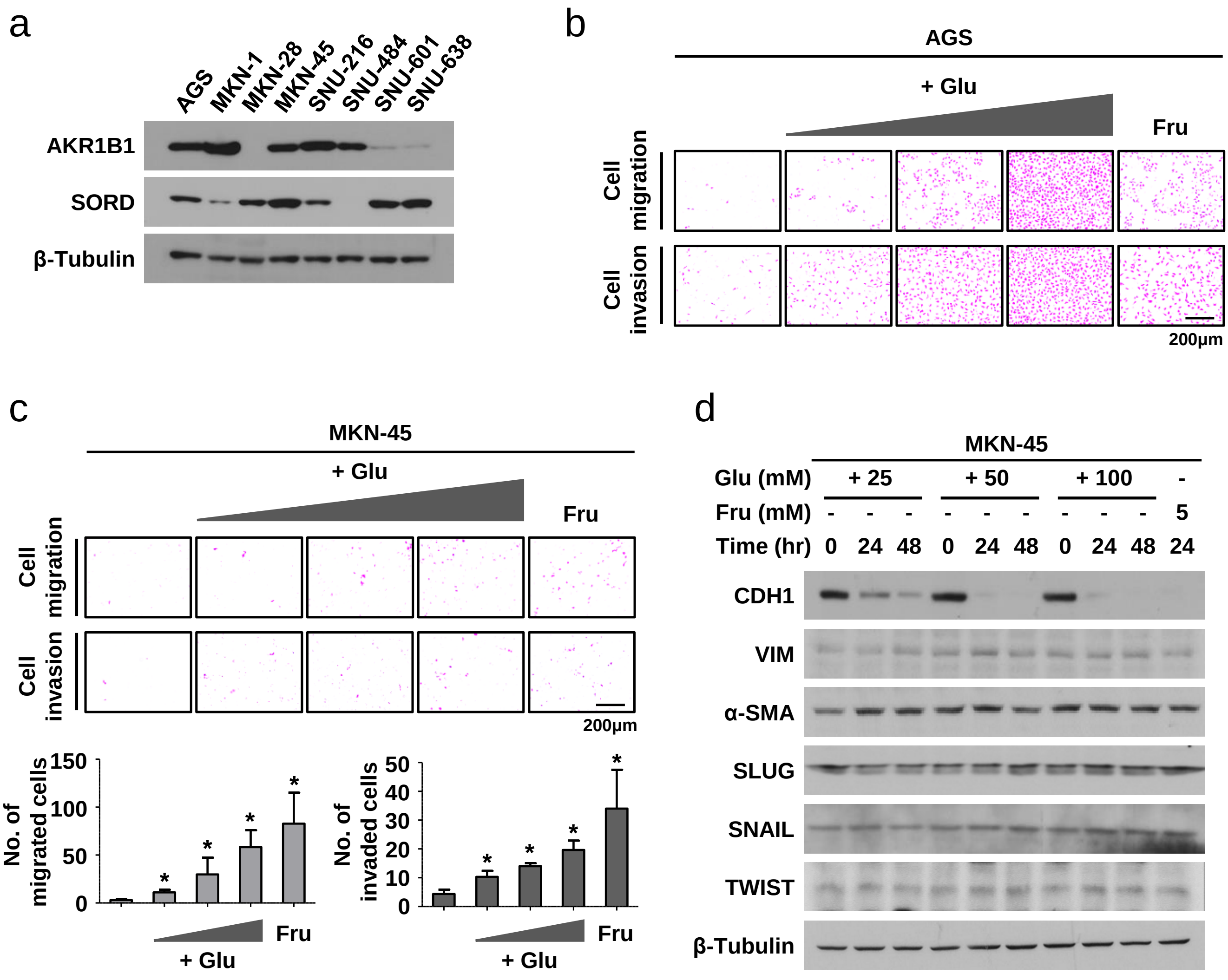

Supplementary Figure 2.

a. Expressions of AKR1B1 and SORD in gastric cancer cell lines. **b.** AGS were incubated with glucose or fructose for 24 hr and subjected to migration and invasion assays. Representative photographs were related with figure 2b. **c.** The migration or invasion potential of MKN-45 was analyzed using Boyden chamber. The cells were incubated in the media supplemented with 25, 50, or 100 mM glucose or 5 mM fructose for 24 hr and placed in the upper chamber. After 24 hr, cells passing though the interface membrane were stained and counted. The numbers (means + SD, $n = 3$) of migrated or invaded cells are presented as bar graphs (left panel) and representative photographs are shown in the top panel. * denotes $P < 0.05$ versus the control group without additional glucose treatment (Student t-test). **d.** MKN-45 cells were incubated with glucose or fructose, and EMT markers were immunoblotted. The blots are representative of three independent experiments.

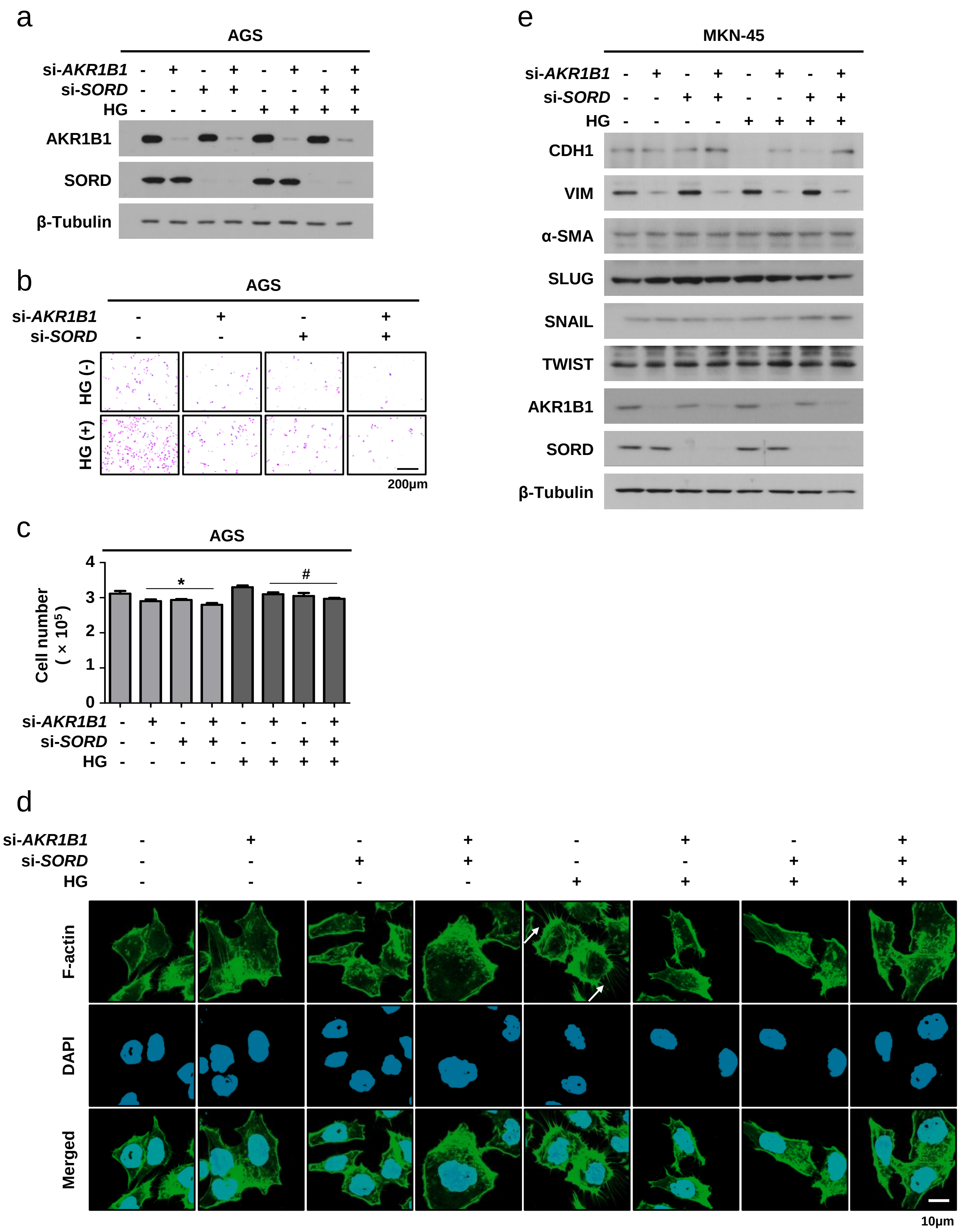
a
AGS
si-AKR1B1
si-SORD
HG
AKR1B1
SORD
β-Tubulin
b
AGS
si-AKR1B1
si-SORD
HG (-)
HG (+)
200μm
c
AGS
Cell number
(× 10^5)
4
3
2
1
0
*
#
si-AKR1B1
si-SORD
HG
d
si-AKR1B1
si-SORD
HG
F-actin
DAPI
Merged
10μm
e
MKN-45
si-AKR1B1
si-SORD
HG
CDH1
VIM
α-SMA
SLUG
SNAIL
TWIST
AKR1B1
SORD
β-Tubulin

Supplementary Figure 3.

a. The gene-silencing efficiency of si-AKR1B1 or si-SORD was verified by immunoblotting in AGS cells. **b.** AGS cells, which had been transfected with si-AKR1B1 and/or si-SORD, were incubated with high glucose (HG) for 24 hr and subjected to migration assay. Representative photographs were related with figure 3a. **c.** AGS cells were transfected with si-AKR1B1 and/or si-SORD, and treated with high glucose (HG) for 24 hr. Cells were seeded at 2×10^5 cells/well in 6-well plates, and counted after 16 hr. The numbers (means + SD, $n = 3$) of cells are presented as bar graphs. *, $P < 0.05$ versus the control group; #, $P < 0.05$ versus the control group with high glucose treatment (Student t-test). **d.** AGS cells were transfected with si-AKR1B1 and/or si-SORD. F-actin and nucleus were stained with Alexa Fluor 488 phalloidin (green) and DAPI (blue), and photographed under fluorescence microscope. Representative photographs were related with figure 3b. **e.** MKN-45 cells, which had been transfected as indicated, were treated with high glucose (HG) for 24 hr. EMT markers were immunoblotted. The blots are representative of three independent experiments.

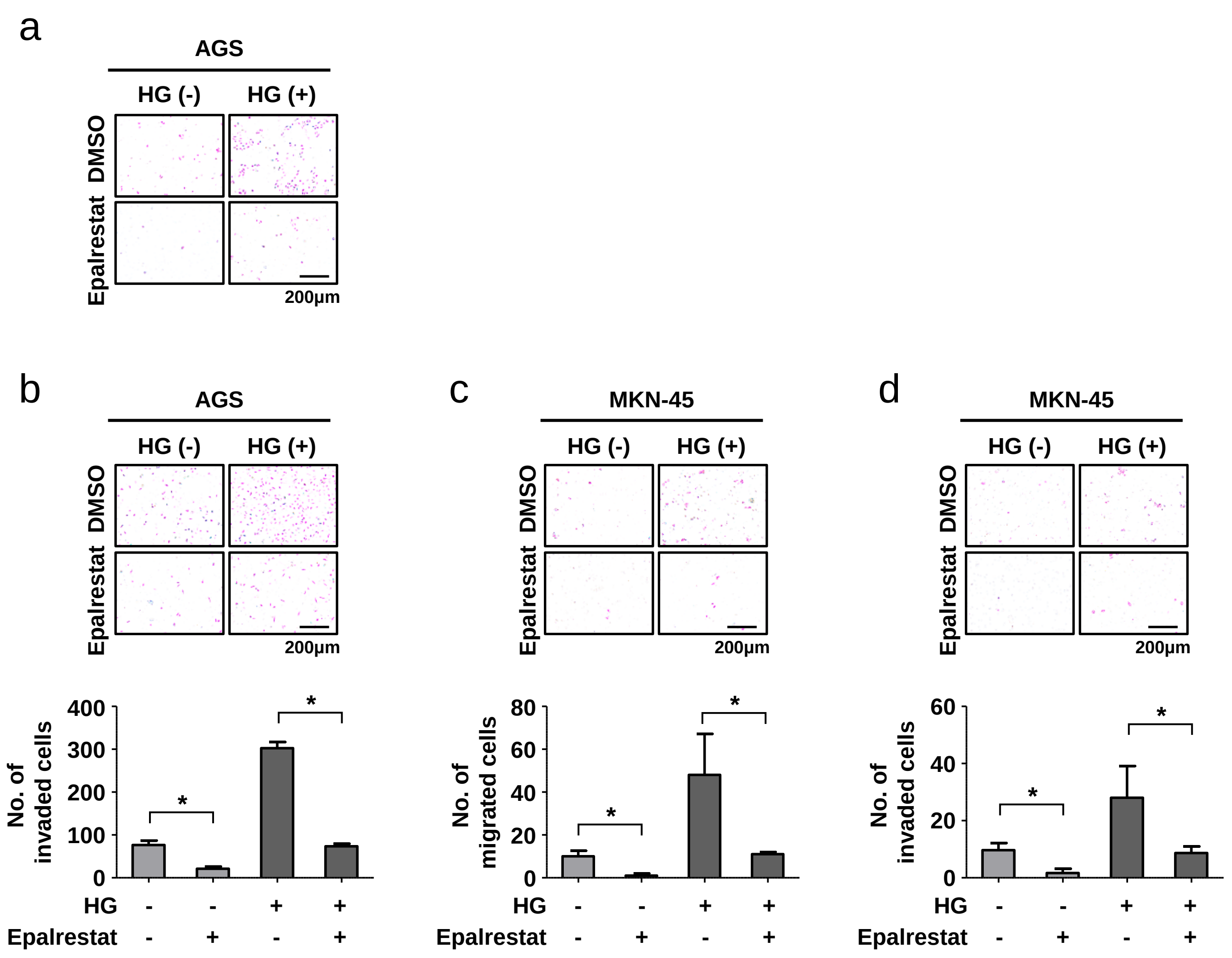

Supplementary Figure 4.

a. AGS cells were treated with high glucose (HG) and 20 μM Epalrestat for 24 hr. Cells were subjected to migration assay. Representative photographs were related with figure 3d. **b-d.** AGS and MKN-45 cells were incubated with high glucose (HG) and 20 μM Epalrestat for 24 hr. Cells are subjected to migration and invasion assays. The numbers (means + SD, n = 3) of migrated or invaded cells are presented as bar graphs (left panel) and representative photographs are shown in the top panel. * denotes $P < 0.05$ between the indicated groups.

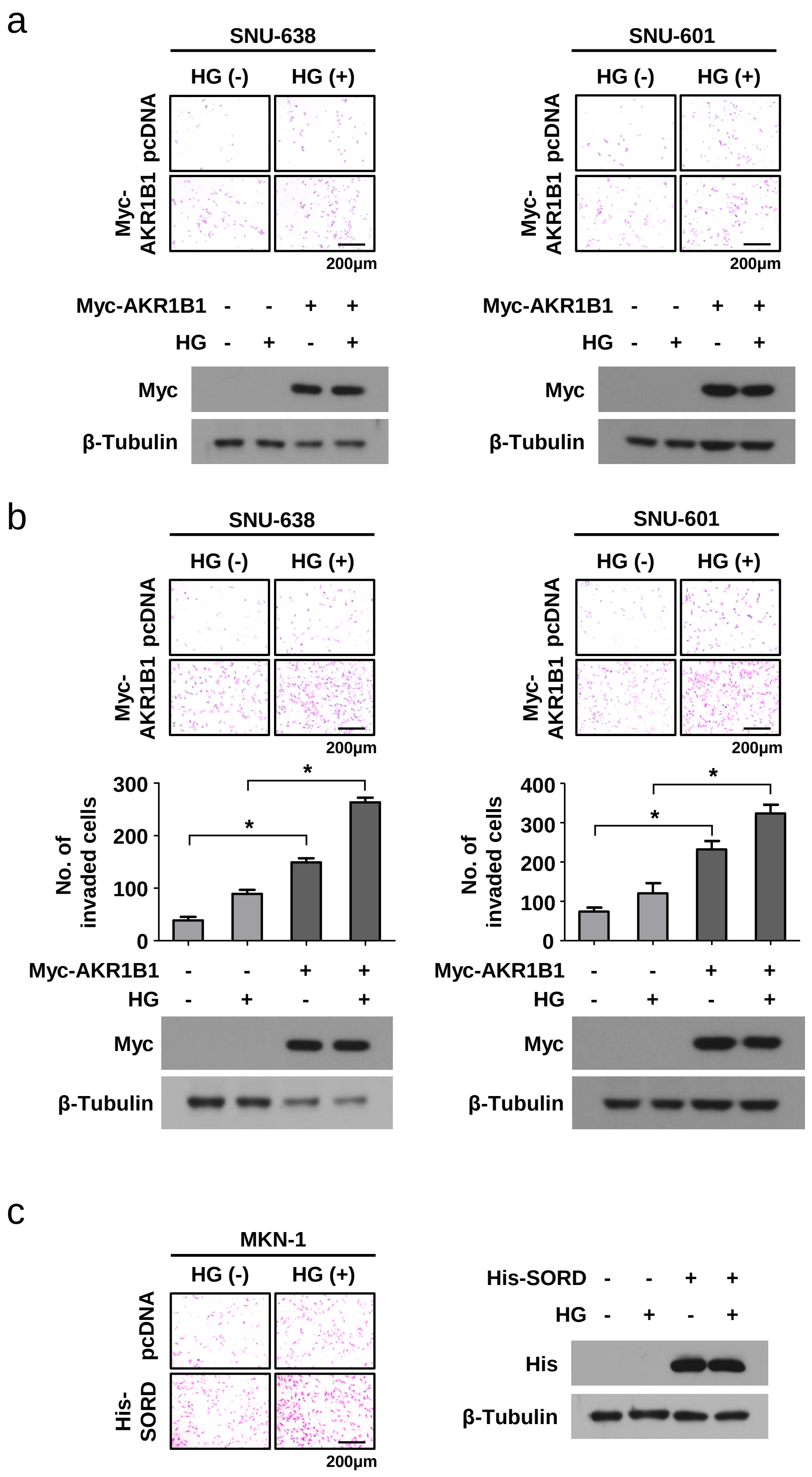
a
SNU-638
HG (-)
HG (+)
pcDNA
Myc-AKR1B1
200μm
SNU-601
Myc-AKR1B1 - - + +
HG - + - +
Myc
β-Tubulin
b
SNU-638
SNU-601
HG (-)
HG (+)
pcDNA
Myc-AKR1B1
200μm
No. of invaded cells
300
200
100
0
400
*
Myc-AKR1B1 - - + +
HG - + - +
Myc
β-Tubulin
c
MKN-1
HG (-)
HG (+)
pcDNA
His-SORD
200μm
His-SORD - - + +
HG - + - +
His
β-Tubulin

Supplementary Figure 5.

a. SNU-638 and SNU-601 cells, which had been transfected with MYC-AKR1B1, were incubated with high glucose (HG) for 24 hr. Cells were subjected to migration assay and transfection efficiency was verified by immunoblotting. Representative photographs were related with figure 3e. **b.** SNU-638 and SNU-601 cells were transfected with MYC-AKR1B1, and incubated with high glucose (HG) for 24 hr. Cells were subjected to invasion assay. The numbers (means + SD, n = 3) of invaded cells are presented as bar graphs (middle panel), and MYC-AKR1B1 expression was verified by Western blotting (bottom panel). Representative photographs are shown. * denotes $P < 0.05$. **c.** MKN-1 cells, which had been transfected with His-SORD, were treated with high glucose (HG) for 24 hr. Cells were subjected to migration assay and immunoblotting. Representative photographs were related with figure 3f.

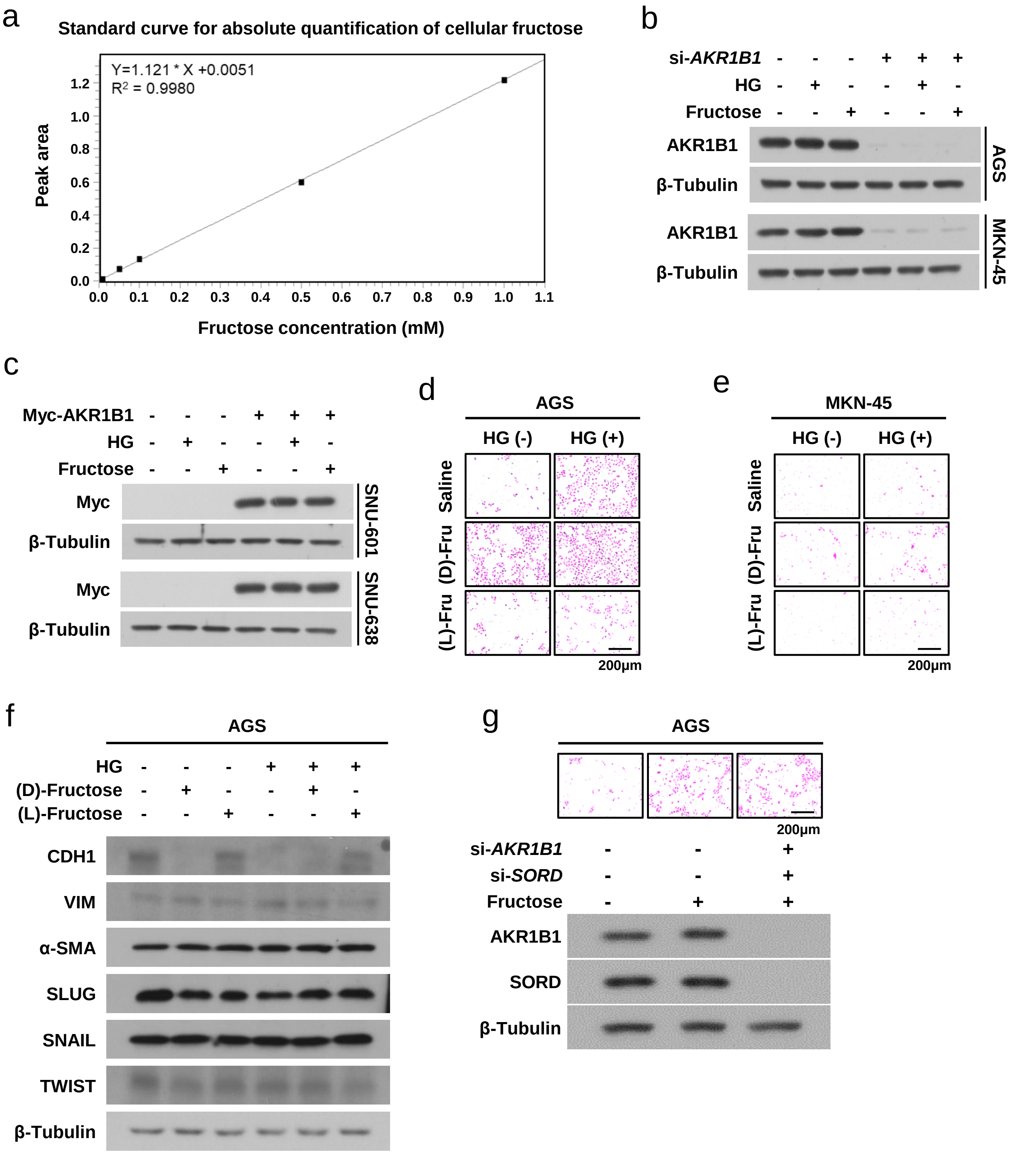
a
Standard curve for absolute quantification of cellular fructose
Y=1.121 * X +0.0051
R2 = 0.9980
Peak area
0.0
0.2
0.4
0.6
0.8
1.0
1.2
0.0 0.1 0.2 0.3 0.4 0.5 0.6 0.7 0.8 0.9 1.0 1.1
Fructose concentration (mM)
b
si-AKR1B1 - - - + + +
HG - + - - + -
Fructose - - + - - +
AKR1B1
β-Tubulin
AGS
AKR1B1
β-Tubulin
MKN-45
c
Myc-AKR1B1 - - - + + +
HG - + - - + -
Fructose - - + - - +
Myc
β-Tubulin
SNU-601
Myc
β-Tubulin
SNU-638
d
AGS
HG (-)
HG (+)
Saline
(D)-Fru
(L)-Fru
200μm
e
MKN-45
HG (-)
HG (+)
Saline
(D)-Fru
(L)-Fru
200μm
f
AGS
HG - - - + + +
(D)-Fructose - + - - + -
(L)-Fructose - - + - - +
CDH1
VIM
α-SMA
SLUG
SNAIL
TWIST
β-Tubulin
g
AGS
200μm
si-AKR1B1 - - +
si-SORD - - +
Fructose - + +
AKR1B1
SORD
β-Tubulin

Supplementary Figure 6.

a. A standard curve of fructose. **b.** AGS and MKN-45 cells were transfected with si-AKR1B1, and incubated with high glucose (HG) or 5 mM fructose for 24 hr. Cells were subjected to LC-MS analysis and transfection efficiency was checked by immunoblotting. **c.** SNU-601 and SNU-638 cells were transfected with MYC-AKR1B1, and treated with high glucose (HG) or 5 mM fructose for 24 hr. Cells were subjected to LC-MS analysis and transfection efficiency was verified by Western blotting. **d-e.** AGS and MKN-45 cells were treated with 5 mM (D)-Fructose, 5 mM (L)-fructose, and/or high glucose (HG) for 24 hr, and subjected to migration assay. Representative photographs were related with figure 4c. **f.** MKN-45 cells were treated with 5 mM (D)-Fructose, 5 mM (L)-fructose, and/or high glucose (HG) for 24 hr, and subjected to Western blotting for EMT markers. The blots are representative of three independent experiments. Representative blots were related with figure 4d. **g.** After transfected AGS cells were incubated with 5 mM fructose for 24 hr, cells are subjected to migration assay and immunoblotting. Representative photographs were related with figure 4e.

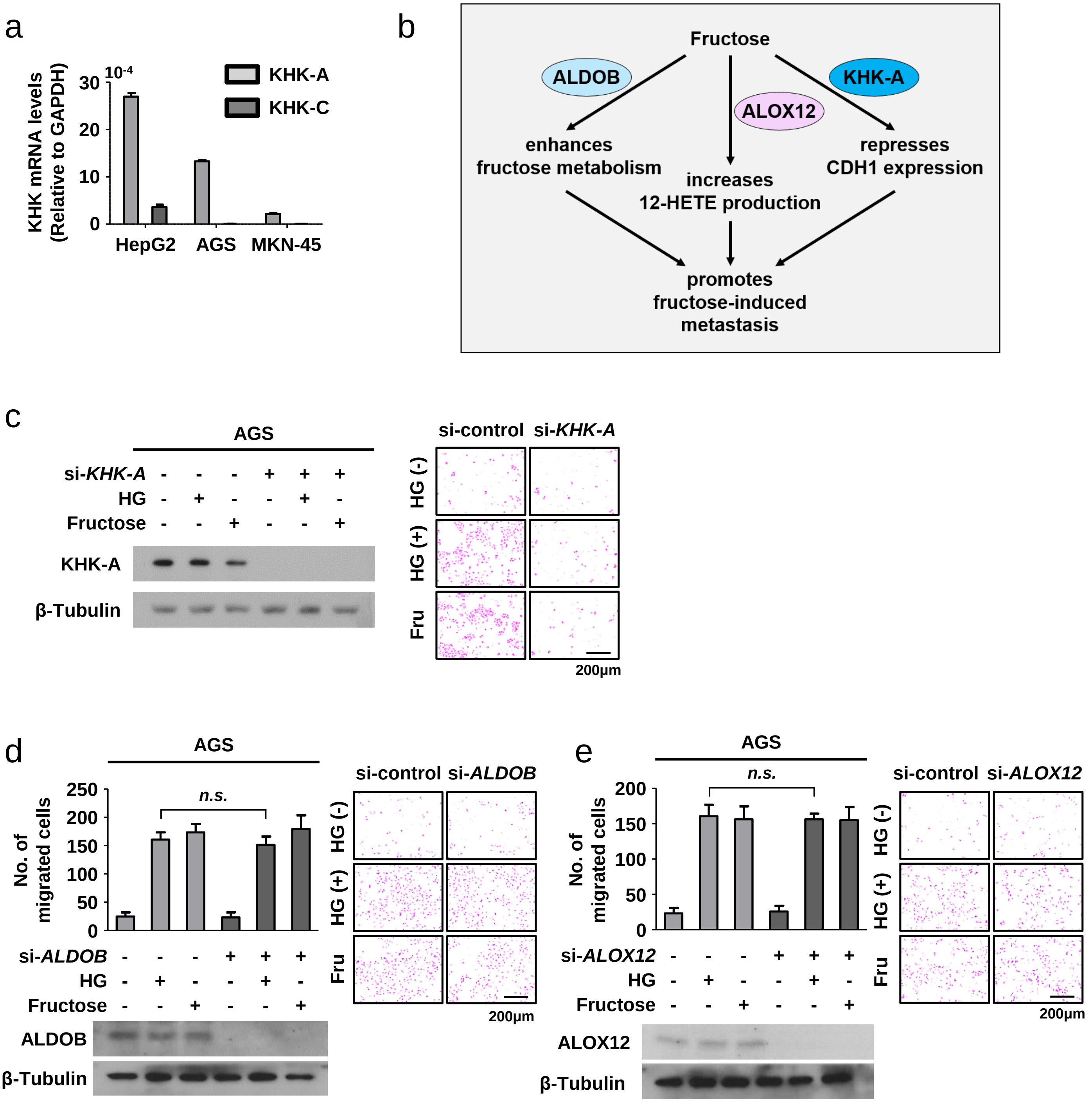
a
KHK mRNA levels (Relative to GAPDH)
10-4
30
20
10
0
KHK-A
KHK-C
HepG2
AGS
MKN-45
b
Fructose
ALDOB
KHK-A
ALOX12
enhances fructose metabolism
increases 12-HETE production
represses CDH1 expression
promotes fructose-induced metastasis
c
AGS
si-KHK-A - - - + + +
HG - + - - + -
Fructose - - + - - +
KHK-A
β-Tubulin
si-control
si-KHK-A
HG (-)
HG (+)
Fru
200μm
d
AGS
No. of migrated cells
250
200
150
100
50
0
n.s.
si-ALDOB - - - + + +
HG - + - - + -
Fructose - - + - - +
ALDOB
β-Tubulin
si-control
si-ALDOB
HG (-)
HG (+)
Fru
200μm
e
AGS
No. of migrated cells
200
150
100
50
0
n.s.
si-ALOX12 - - - + + +
HG - + - - + -
Fructose - - + - - +
ALOX12
β-Tubulin
si-control
si-ALOX12
HG (-)
HG (+)
Fru
200μm

Supplementary Figure 7.

a. The mRNA levels (means + SD, n = 3) of KHK-A and KHK-C were measured by quantitative RT-PCR. **b.** Previously proposed pathways related to fructose-induced cancer progression. **c**. AGS cells, which had been transfected with 80 nM si-KHK-A, were incubated with high glucose (HG) or 5 mM fructose for 24 hr. Cells were subjected to migration assay and transfection efficiency was checked by immunoblotting. Representative photographs were related with figure 5a. **d-e**. AGS cells, which had been transfected with 80 nM ALDOB si-RNA or 80 nM si-ALOX12, were incubated with high glucose (HG) or 5 mM fructose for 24 hr. Cells were subjected to migration assay, and the numbers (means + SD, *n* = 3) of migrated cells are presented as bar graphs (left panel). The efficiencies of si-RNAs were verified by Western blotting and representative photographs are shown. *n.s.*, not significant difference.

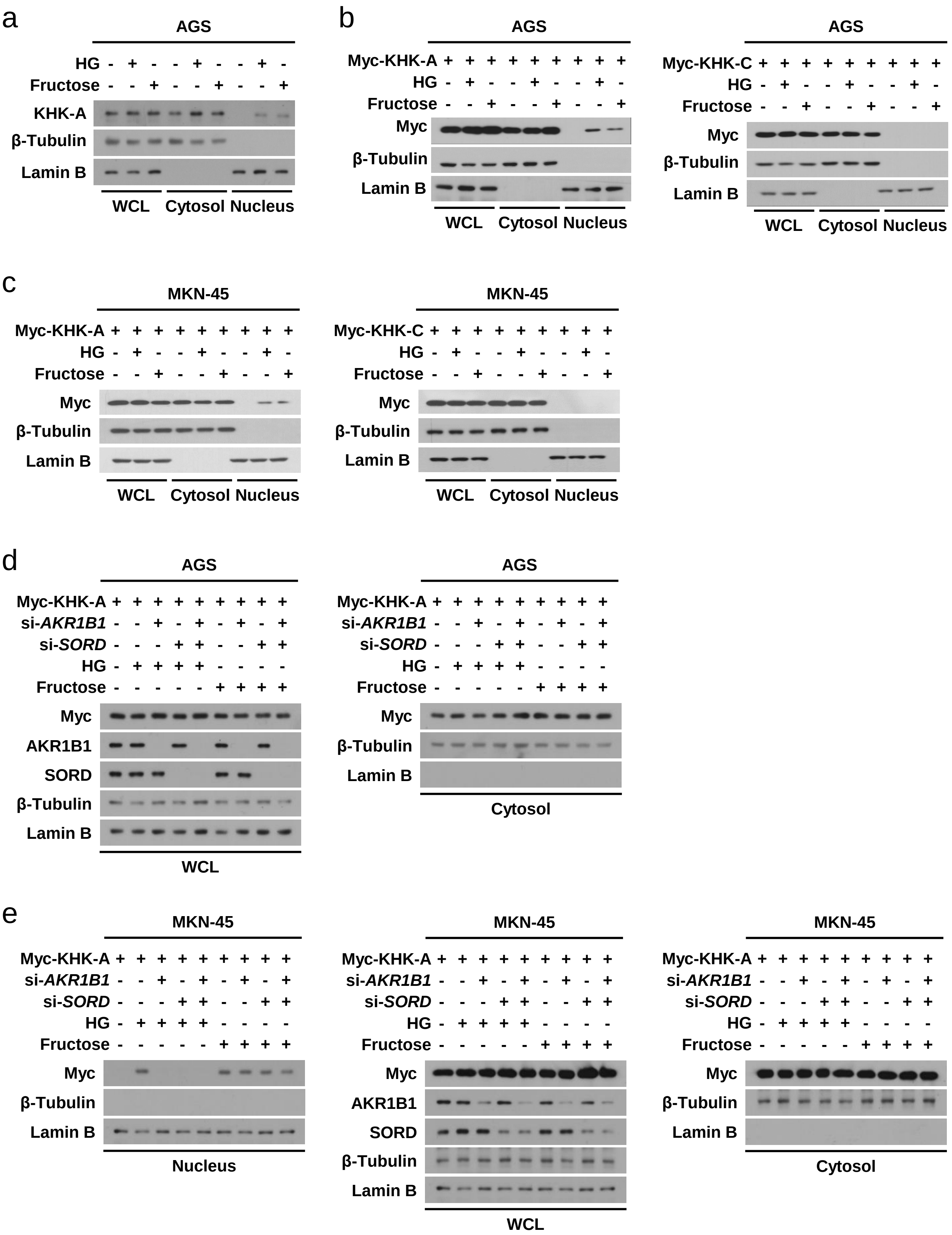
a
AGS
HG - + - - + - - + -
Fructose - - + - - + - - +
KHK-A
β-Tubulin
Lamin B
WCL
Cytosol
Nucleus
b
AGS
Myc-KHK-A + + + + + + + + +
HG - + - - + - - + -
Fructose - - + - - + - - +
Myc
β-Tubulin
Lamin B
WCL
Cytosol
Nucleus
AGS
Myc-KHK-C + + + + + + + + +
HG - + - - + - - + -
Fructose - - + - - + - - +
Myc
β-Tubulin
Lamin B
WCL
Cytosol
Nucleus
c
MKN-45
Myc-KHK-A + + + + + + + + +
HG - + - - + - - + -
Fructose - - + - - + - - +
Myc
β-Tubulin
Lamin B
WCL
Cytosol
Nucleus
MKN-45
Myc-KHK-C + + + + + + + + +
HG - + - - + - - + -
Fructose - - + - - + - - +
Myc
β-Tubulin
Lamin B
WCL
Cytosol
Nucleus
d
AGS
Myc-KHK-A + + + + + + + + +
si-AKR1B1 - - + - + - + - +
si-SORD - - - + + - - + +
HG - + + + + - - - -
Fructose - - - - - + + + +
Myc
AKR1B1
SORD
β-Tubulin
Lamin B
WCL
AGS
Myc-KHK-A + + + + + + + + +
si-AKR1B1 - - + - + - + - +
si-SORD - - - + + - - + +
HG - + + + + - - - -
Fructose - - - - - + + + +
Myc
β-Tubulin
Lamin B
Cytosol
e
MKN-45
Myc-KHK-A + + + + + + + + +
si-AKR1B1 - - + - + - + - +
si-SORD - - - + + - - + +
HG - + + + + - - - -
Fructose - - - - - + + + +
Myc
β-Tubulin
Lamin B
Nucleus
MKN-45
Myc-KHK-A + + + + + + + + +
si-AKR1B1 - - + - + - + - +
si-SORD - - - + + - - + +
HG - + + + + - - - -
Fructose - - - - - + + + +
Myc
AKR1B1
SORD
β-Tubulin
Lamin B
WCL
MKN-45
Myc-KHK-A + + + + + + + + +
si-AKR1B1 - - + - + - + - +
si-SORD - - - + + - - + +
HG - + + + + - - - -
Fructose - - - - - + + + +
Myc
β-Tubulin
Lamin B
Cytosol

Supplementary Figure 8.

a. AGS cells were incubated with high glucose (HG) or 5 mM fructose for 24 hr. Whole cell lysates (WCL) were fractionated to cytosolic and nuclear components and subjected to immunoblotting. **b.** After AGS cells expressing Myc-KHK-A or Myc-KHK-C were treated with high glucose (HG) or 5 mM fructose for 24 hr, whole cell lysates were fractionated to cytosolic and nuclear components. Myc-KHK-A and Myc-KHK-C were immunoblotted in subcellular components. **c.** MKN-45 cells expressing Myc-KHK-A or Myc-KHK-C were incubated with high glucose (HG) or 5 mM fructose for 24 hr. KHK-A and KHK-C were immunoblotted in the nuclear fraction. **d.** AGS cells, which had been transfected as indicated, were treated with high glucose (HG) or 5 mM fructose for 24 hr. Myc-KHK-A was immunoblotted in subcellular components. **e.** After transfected MKN-45 cells were incubated with high glucose (HG) or fructose for 24 hr, cell lysates were fractionated to the cytosolic and nuclear fractions, and subjected to immunoblotting.

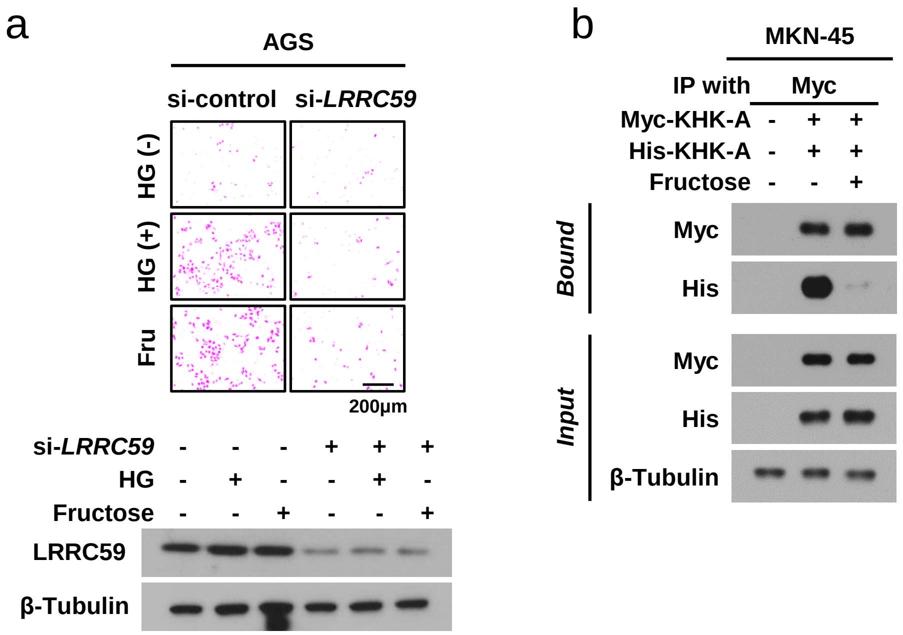

Supplementary Figure 9.

a. AGS cells, which had been transfected with si-LRRC59, were treated with high glucose (HG) or 5 mM fructose for 24 hr. Cells were subjected to migration assay and Western blotting. Representative photographs are related with figure 5f. **b.** MKN-45 cells were transfected as indicated, and incubated 5 mM fructose for 24 hr. The cell lysates were immunoprecipitated and immunoblotted.

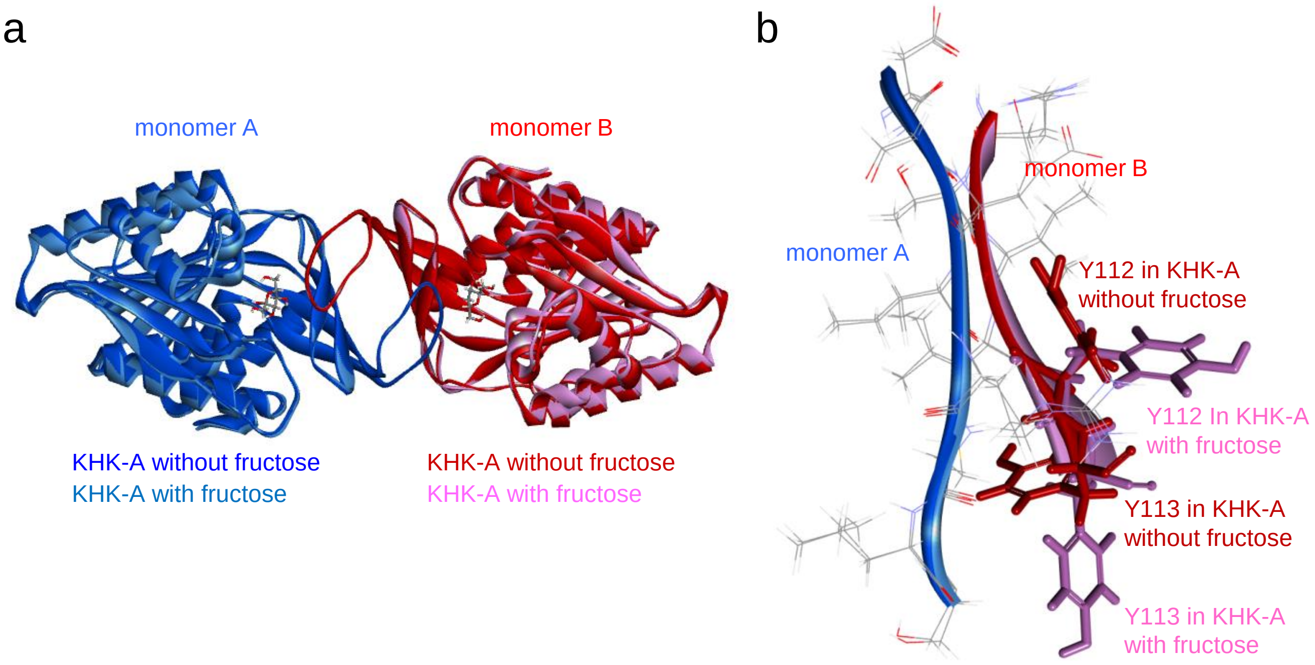

Supplementary Figure 10.

Superimposed configurations of fructose-free and fructose-bound KHK-A configurations: **a.** Entire KHK-A backbones with fructose-free shown in red and blue, and fructose-bound in pink and light blue. **b.** Detailed overlay at the monomer-monomer interface, highlighting Asp27-Ser34 for monomer A and Arg108-Tyr113 for monomer B. Side chains maintaining consistent orientations are represented by thin lines, whereas those with altered orientations are depicted as sticks.

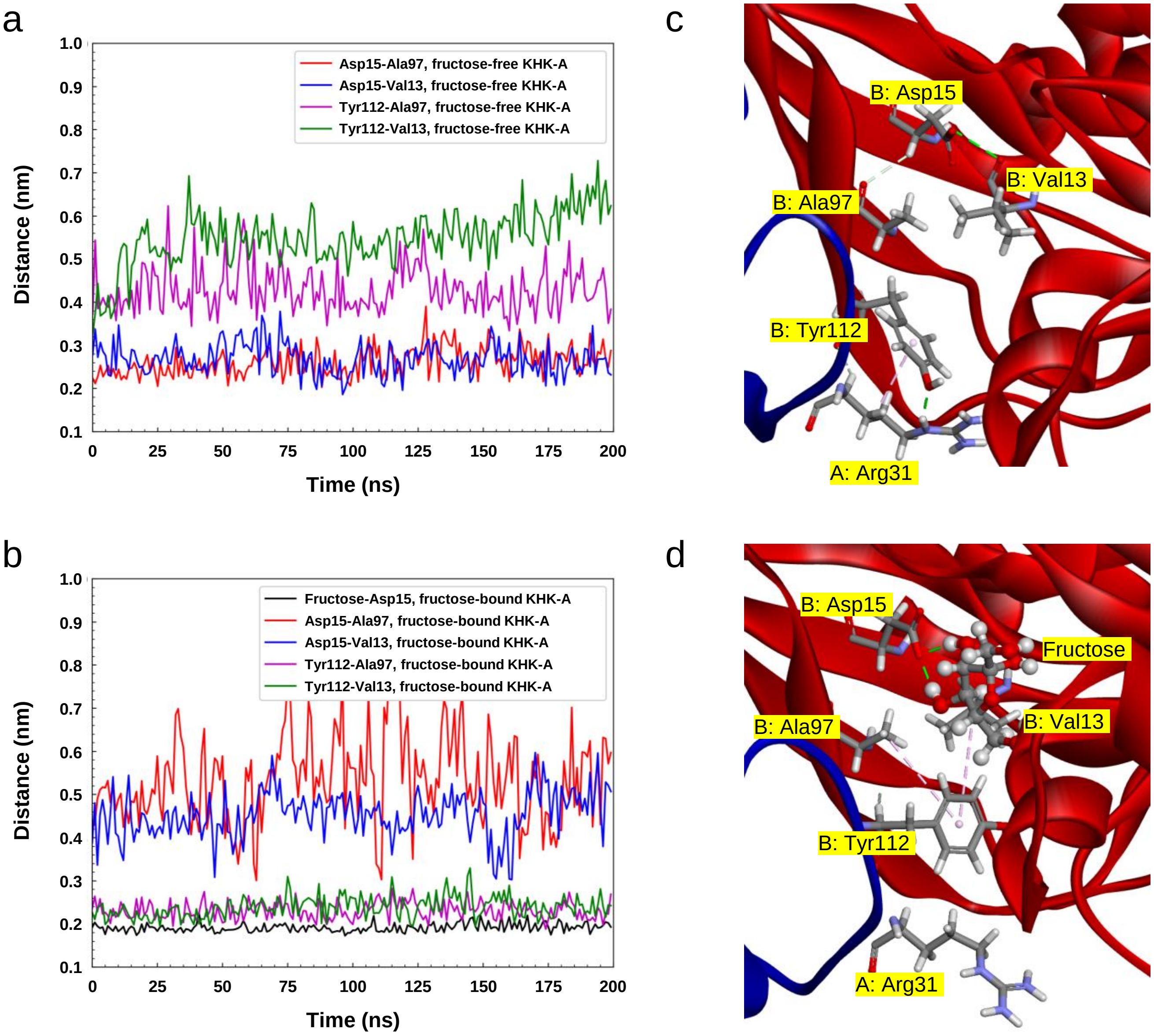

Supplementary Figure 11.

Variation in minimum distances between residues involving fructose sensing over MD simulation time: (**a**) for fructose-free KHK-A and (**b**) for fructose-bound KHK-A. Structural representations of (**c**) fructose-free KHK-A and (**d**) fructose-bound KHK-A focusing on the region containing the residues. In these structures, selected residues are depicted as sticks, while the fructose in (**d**) is shown as ball-and-stick.

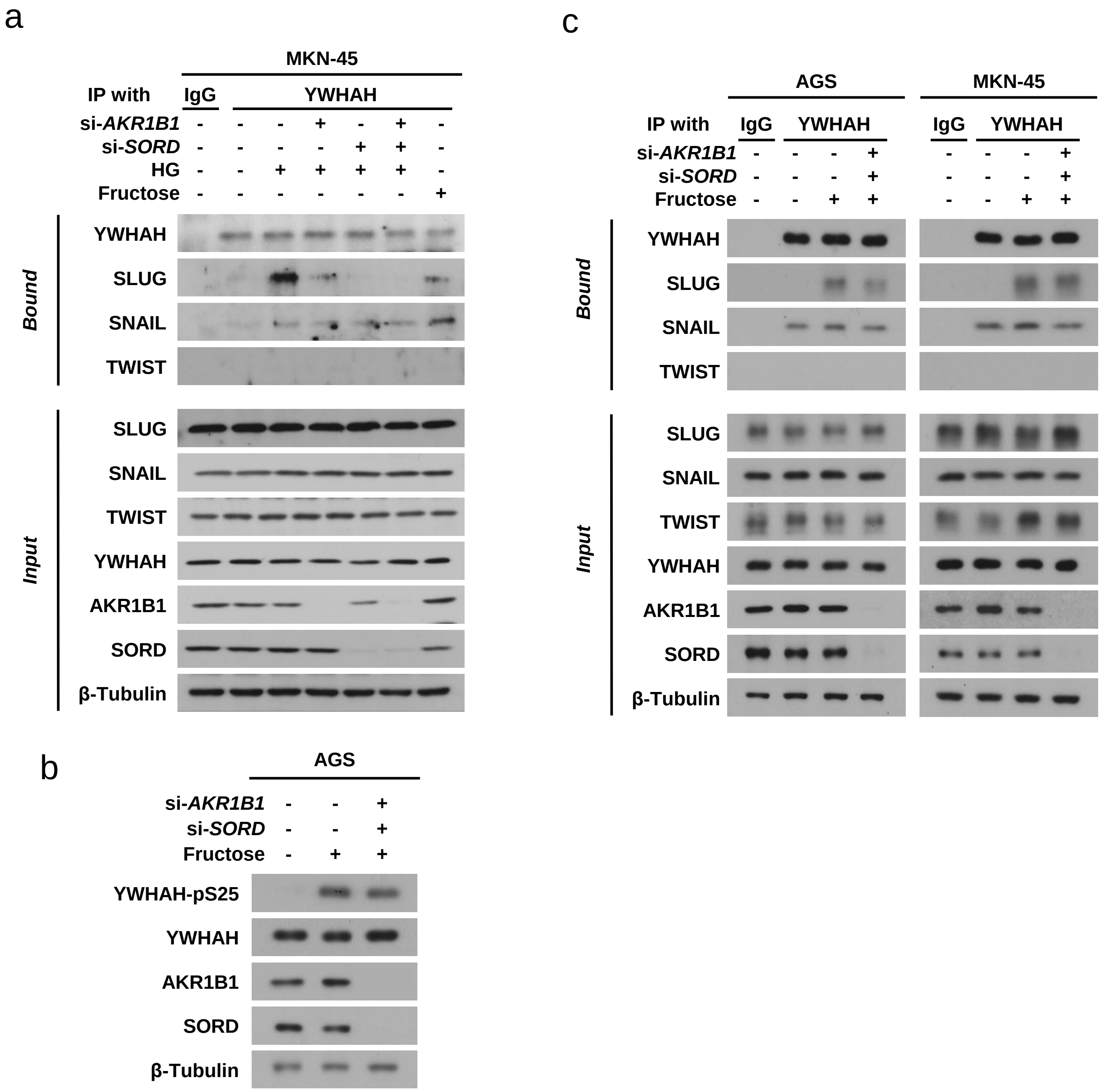

Supplementary Figure 12.

a. YWHAH binding to endogenous SLUG, SNAIL, or TWIST. MKN-45 cells, which had been transfected with si-AKR1B1 and/or si-SORD, were treated with high glucose (HG) or 5 mM fructose for 24 hr. Cells were immunoprecipitated with IgG or anti-YWHAH, and then immunoblotted. **b.** In transfected AGS cells treated with fructose for 24 hr, YWHAH at Ser25 was evaluated by immunoblotting. **c**. Transfected AGS and MKN-45 cells were incubated as indicated, and subjected to immunoprecipitation and immunoblotting.

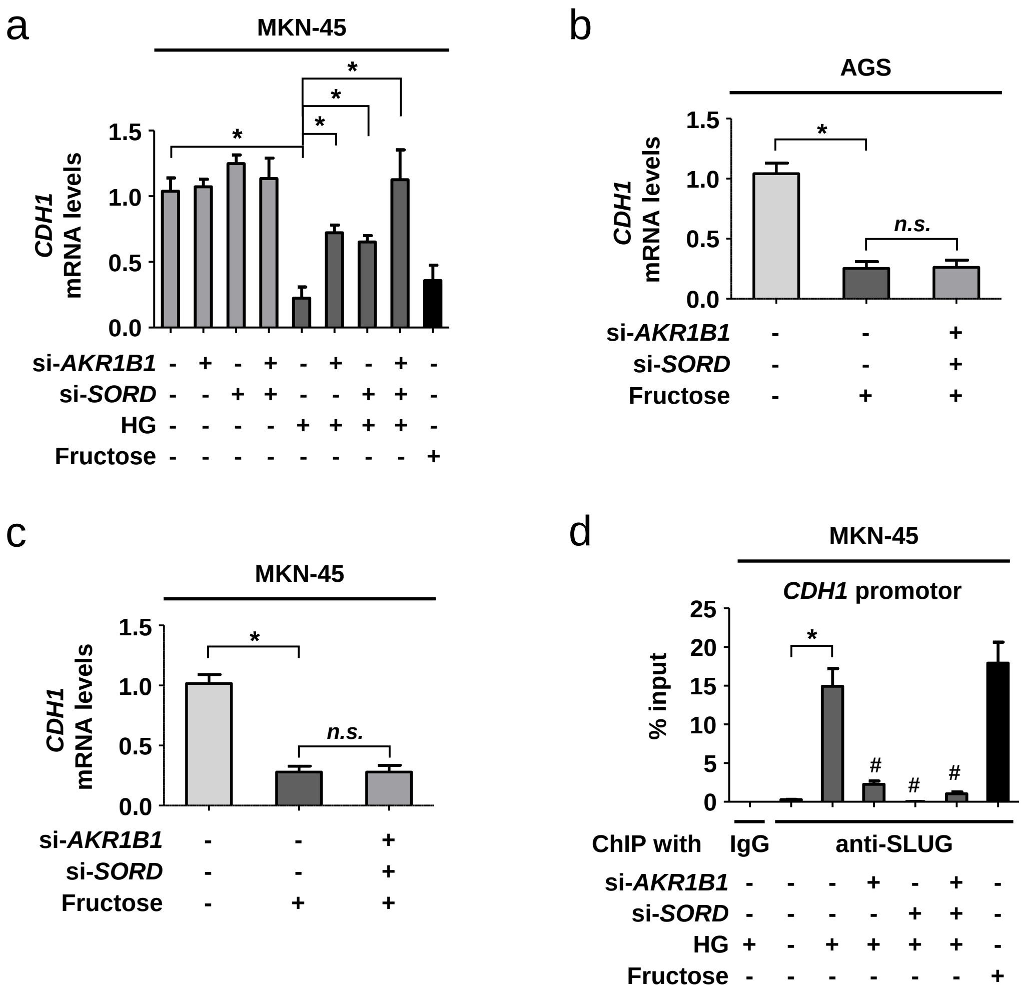

Supplementary Figure 13.

a. The mRNA levels of CDH1 were analyzed by RT-qPCR in MKN-45 cells. MKN-45 cells, which had been transfected as indicated, were incubated with high glucose (HG) or 5 mM fructose for 24 hr. Each bar represents the mean + SD (n = 3). **b-c.** In AGS and MKN-45 cells, the mRNA levels (the mean + SD, n = 3) of CDH1 were measured by RT-qPCR (means + SD, n = 3). Transfected AGS and MKN-45 cells were treated with 5 mM fructose for 24 hr. *, $P < 0.05$; *n.s.*, not significant. **d.** MKN-45 cells were transfected with si-AKR1B1, and/or si-SORD. After incubated with high glucose (HG) or 5 mM fructose for 24 hr, cells were subjected to ChIP-qPCR using anti-SLUG antibody. *, $P < 0.05$ versus the si-control group; #, $P < 0.05$ versus the si-control treated with glucose group.

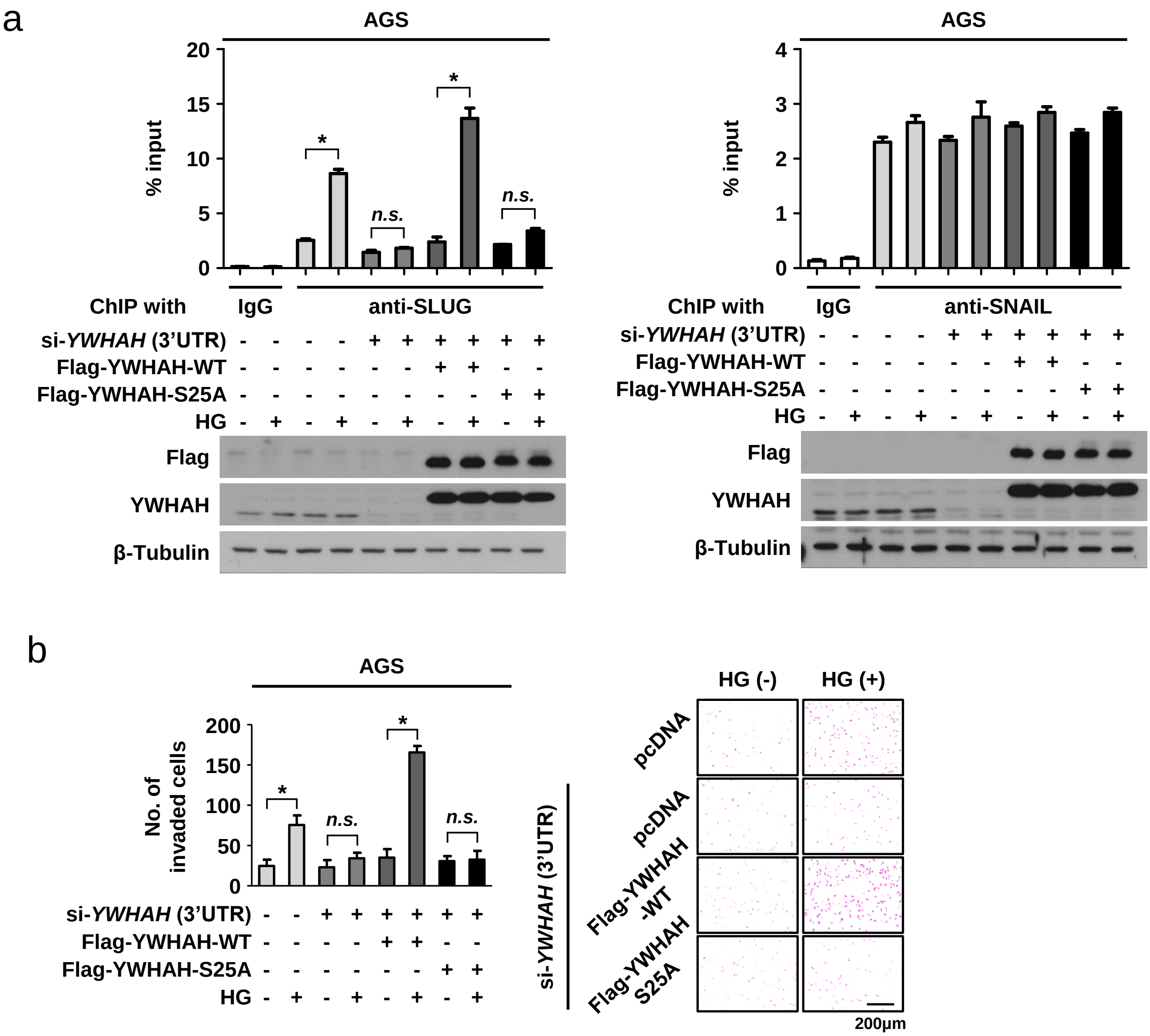

Supplementary Figure 14.

a. Phosphorylated YWHAH is essential for the SLUG binding to the CDH1 promotor. AGS cells, which had been transfected as indicated, were incubated with high glucose (HG) for 24 hr. Cells were subjected to ChIP-qPCR using anti-SLUG antibody or anti-SNAIL antibody. Results (the mean + SD, n = 3) were presented as the percentages of input data. **b.** The invasion potential of AGS cells was analyzed using Boyden chamber with Matrigel-coated membrane. AGS cells, which had been transfected as indicated, were treated with high glucose (HG) for 24 hr. The numbers (means + SD, *n* = 3) of invaded cells are presented as bar graphs (left panel) and representative photographs are shown in the right panel. * denotes $P < 0.05$ between the indicated groups; *n.s.*, not significantly different.

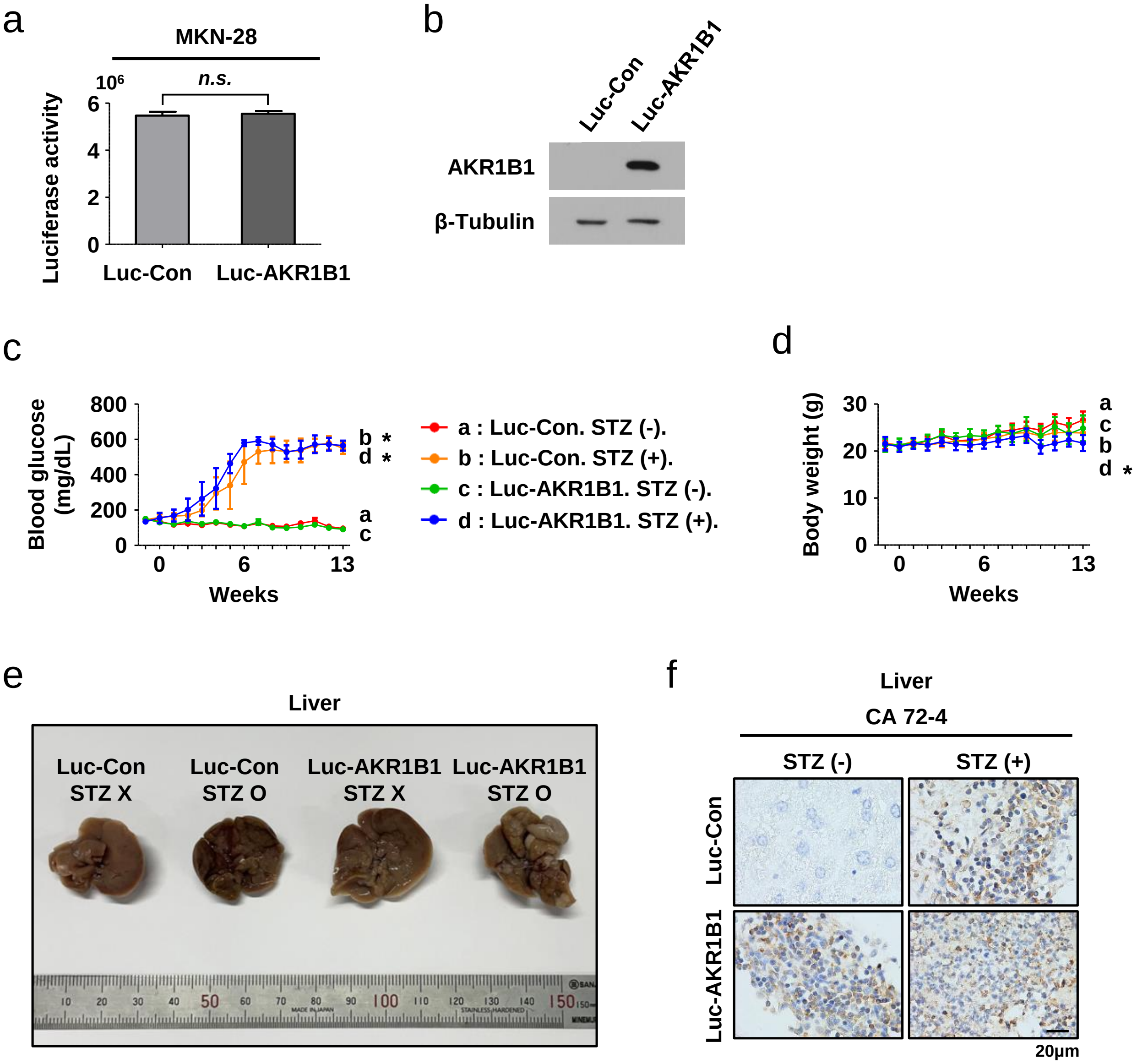

Supplementary Figure 15.

a. Establishment of stable cells. MKN-28 cells were transfected with the Luciferase-IRES-GFP or the Luciferase-IRES-AKR1B1 and selected with G418. The luciferase activity was confirmed by luminometry. *n.s.*, not significant difference. **b.** The expression of AKR1B1 of stable cells was checked by immunoblotting. **c.** The blood glucose levels of mice were checked once a week. Conditions for each experimental group (7 per each group) are described in the right panel. **d.** Body weights of mice (the means ± S.D. from 7 mice per group). * denotes $P < 0.05$ versus the group ‘a’. **e.** Representative photographs showing metastatic tumors on livers. **f.** Representative photographs of liver tissues immunostained with the antibody against CA 72-4.

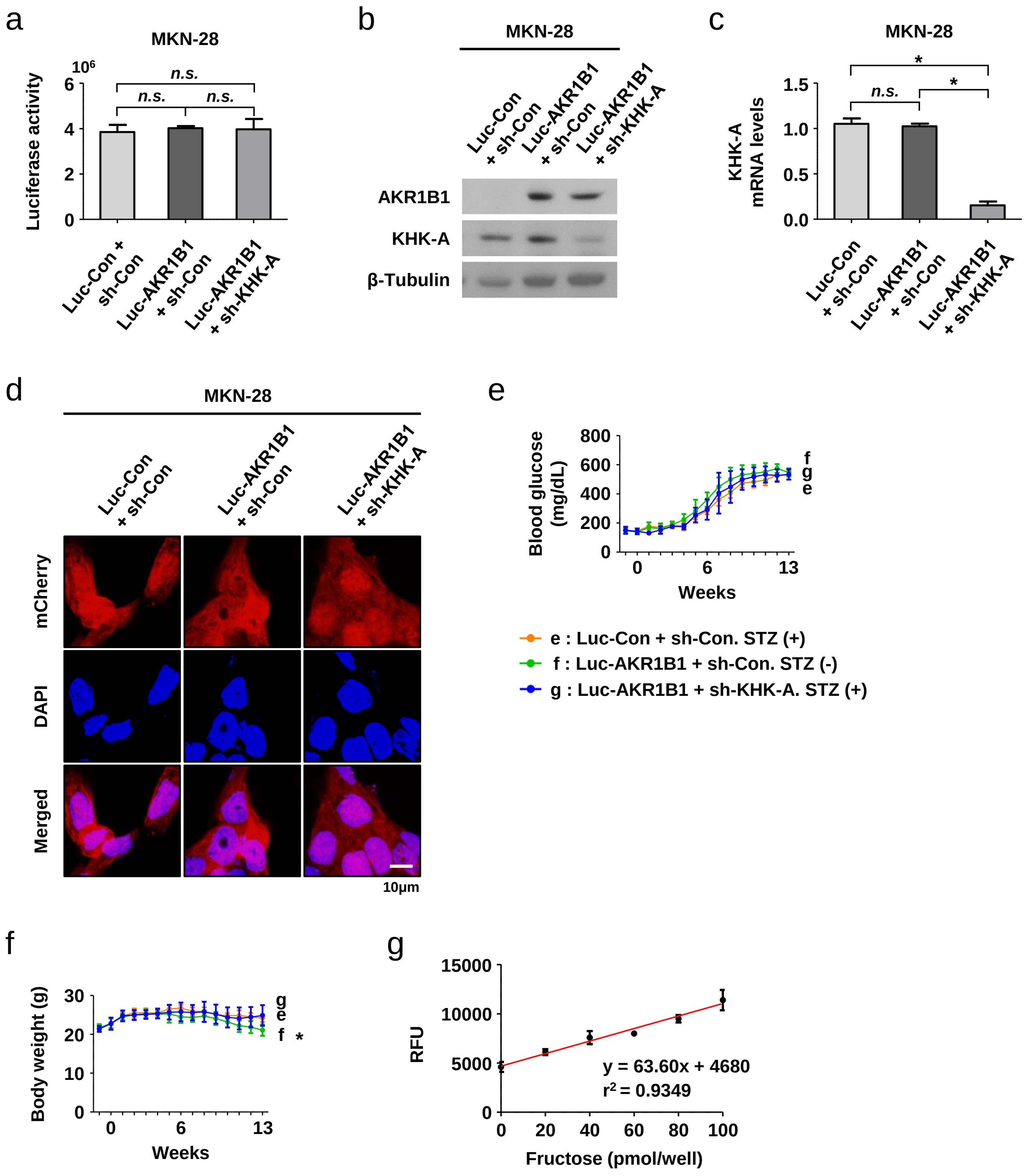

a
MKN-28
Luciferase activity
10^6
n.s.
n.s.
n.s.
Luc-Con + sh-Con
Luc-AKR1B1 + sh-Con
Luc-AKR1B1 + sh-KHK-A
b
MKN-28
Luc-Con + sh-Con
Luc-AKR1B1 + sh-Con
Luc-AKR1B1 + sh-KHK-A
AKR1B1
KHK-A
β-Tubulin
c
MKN-28
KHK-A mRNA levels
n.s.
*
*
Luc-Con + sh-Con
Luc-AKR1B1 + sh-Con
Luc-AKR1B1 + sh-KHK-A
d
MKN-28
Luc-Con + sh-Con
Luc-AKR1B1 + sh-Con
Luc-AKR1B1 + sh-KHK-A
mCherry
DAPI
Merged
10μm
e
Blood glucose (mg/dL)
Weeks
f
g
e
e : Luc-Con + sh-Con. STZ (+)
f : Luc-AKR1B1 + sh-Con. STZ (-)
g : Luc-AKR1B1 + sh-KHK-A. STZ (+)
f
Body weight (g)
Weeks
g
e
f *
g
RFU
Fructose (pmol/well)
y = 63.60x + 4680
r^2 = 0.9349

Supplementary Figure 16.

a. Establishment of stable cells. MKN-28 cells were transfected with the Luciferase-IRES-GFP or Luciferase-IRES-AKR1B1, and the cells were also infected with lentiviral shRNA targeting KHK-A (sh-KHK-A) or a scrambled sequence (sh-Con). Transfected cells were selected with G418 and puromycin. The expression of luciferase was confirmed by luminometry. *n.s.*, not significantly different. **b.** The expression of AKR1B1 and KHK-A were checked by Western blotting. **c.** Silencing of endogenous KHK-A was confirmed by RT-qPCR. *, $P < 0.05$; *n.s.*, not significant. **d.** Successful infection with mCherry-expressing lentiviral shRNA plasmid was checked by immunofluorescence. **e.** Blood glucose levels of mice. Conditions for each experimental group (7 per each group) are described in the bottom panel. **f.** Body weights of mice were checked once a week. * denotes $P < 0.05$ versus the group 'e'. **g.** Fructose standard curve in the fructose assay kit.

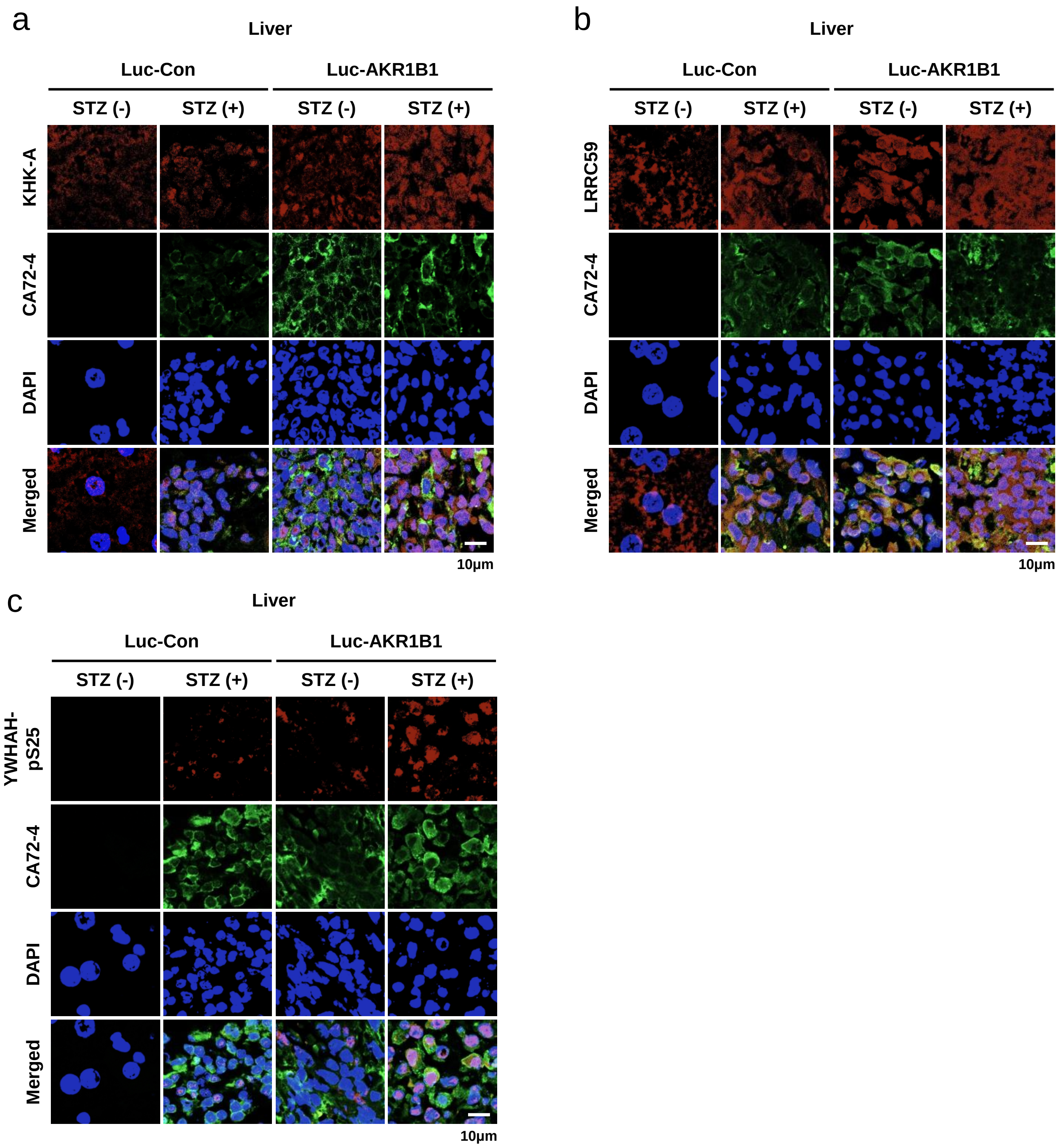

Supplementary Figure 17.

Representative immunofluorescence images related with figure 7h. The excised livers were stained with the indicated antibodies and anti-CA 72-4 antibody. KHK-A (**a**), LRRC59 (**b**), YWHAH-pS25 (**c**) were visualized by Alexa Flour 594-conjugated secondary antibodies (red), and CA 72-4 were examined by Alexa Flour 488-conjugated secondary antibodies (green). Nuclei were stained with DAPI (blue).

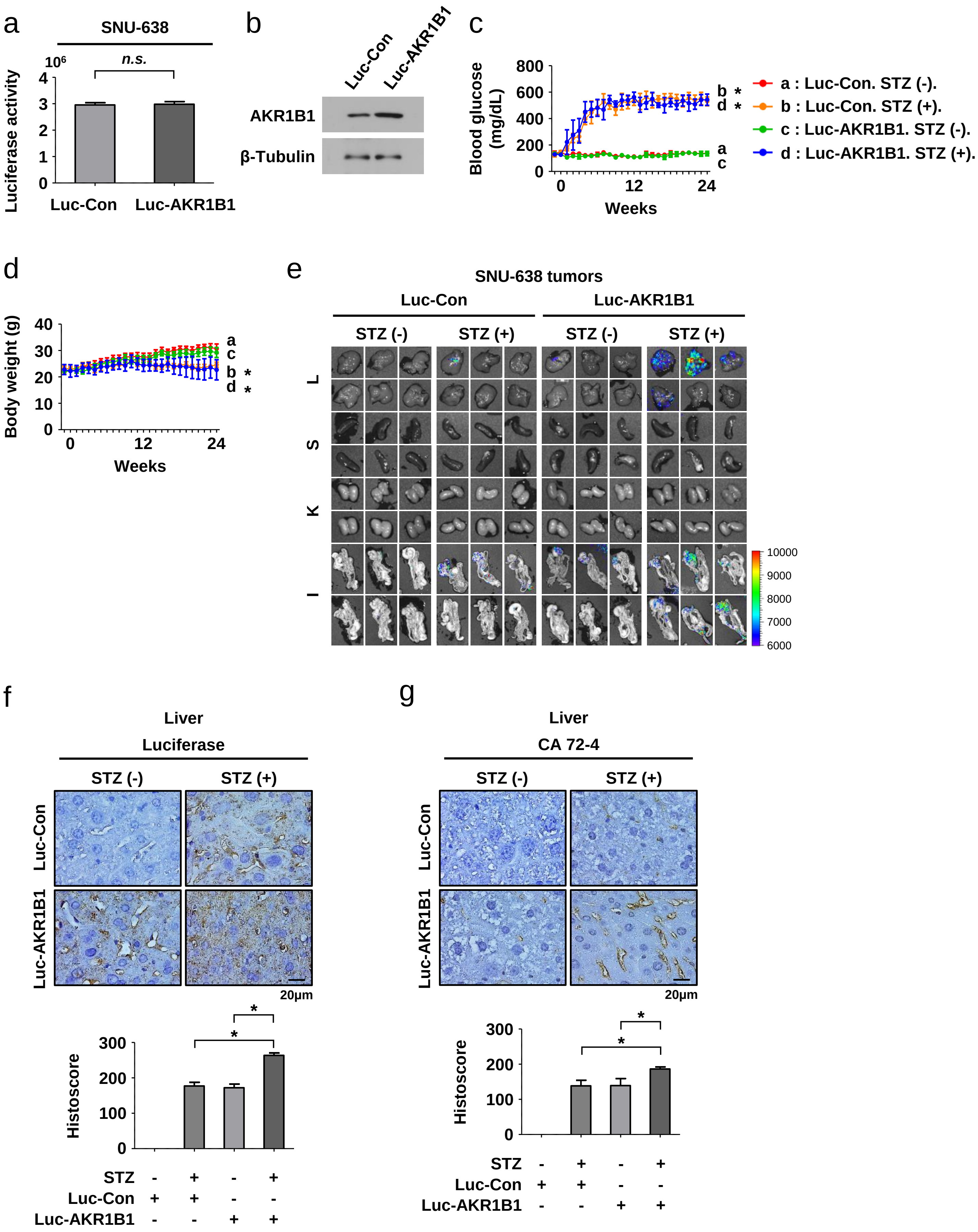

a
SNU-638
n.s.
Luciferase activity
10^6
Luc-Con
Luc-AKR1B1
b
Luc-Con
Luc-AKR1B1
AKR1B1
β-Tubulin
c
Blood glucose (mg/dL)
Weeks
a : Luc-Con. STZ (-).
b : Luc-Con. STZ (+).
c : Luc-AKR1B1. STZ (-).
d : Luc-AKR1B1. STZ (+).
d
Body weight (g)
Weeks
e
SNU-638 tumors
Luc-Con
Luc-AKR1B1
STZ (-)
STZ (+)
L
S
K
I
f
Liver
Luciferase
STZ (-)
STZ (+)
Luc-Con
Luc-AKR1B1
20μm
Histoscore
STZ
Luc-Con
Luc-AKR1B1
g
Liver
CA 72-4
STZ (-)
STZ (+)
Luc-Con
Luc-AKR1B1
20μm
Histoscore
STZ
Luc-Con
Luc-AKR1B1

Supplementary Figure 18.

a. Establishment of stable cells. SNU-638 cells were transfected with the Luciferase-IRES-GFP or the Luciferase-IRES-AKR1B1 and selected with G418. The expression of luciferase was checked by luminometry. *n.s.*, not significant difference. **b.** The expression of AKR1B1 was confirmed by immunoblotting. **c.** Blood glucose levels of mice. Conditions for each experimental group (6 per each group) are described in the right panel. **d.** Body weights of mice were monitored once a week. *, $P < 0.05$ versus the group 'a'. **e.** Bioluminescence images of organs excised from mice were captured using Xenogen IVIS 100, related to Figure 8d. **f-g.** Representative photographs of liver tissues immunostained with anti-luciferase antibody (**f**) and anti-CA 72-4 antibody (**g**) (top panel). The expression levels were evaluated using histoscore and presented as bar graphs (bottom panel). * denotes $P < 0.05$ between the indicated groups.

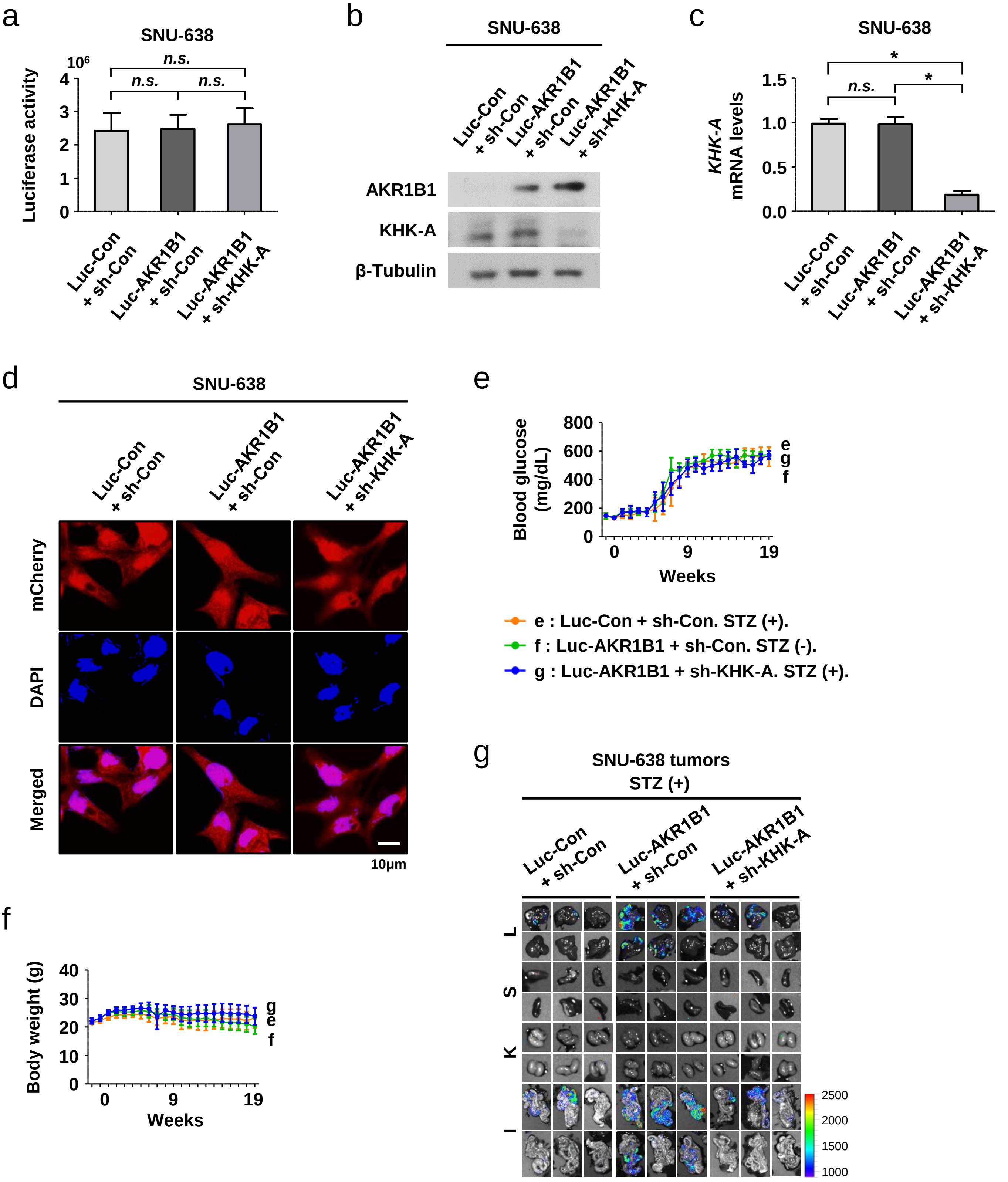
a
SNU-638
10^6
Luciferase activity
n.s.
n.s.
n.s.
Luc-Con + sh-Con
Luc-AKR1B1 + sh-Con
Luc-AKR1B1 + sh-KHK-A
b
SNU-638
Luc-Con + sh-Con
Luc-AKR1B1 + sh-Con
Luc-AKR1B1 + sh-KHK-A
AKR1B1
KHK-A
β-Tubulin
c
SNU-638
KHK-A mRNA levels
n.s.
*
*
d
SNU-638
mCherry
DAPI
Merged
10μm
e
Blood glucose (mg/dL)
Weeks
e : Luc-Con + sh-Con. STZ (+).
f : Luc-AKR1B1 + sh-Con. STZ (-).
g : Luc-AKR1B1 + sh-KHK-A. STZ (+).
f
Body weight (g)
Weeks
g
SNU-638 tumors
STZ (+)
L
S
K
I
2500
2000
1500
1000

Supplementary Figure 19.

a. Establishment of stable cells. SNU-638 cells, which had been transfected with the Luciferase-IRES-GFP or Luciferase-IRES-AKR1B1, and also infected with lentiviral shRNA targeting KHK-A or a scrambled sequence (sh-Con), were selected with G418 and puromycin. The expression of luciferase was confirmed by luminometry. **b.** The expression of AKR1B1 and KHK-A were checked by immunoblotting. **c.** The gene-silencing efficacy of lentiviral shRNA targeting KHK-A was evaluated by RT-qPCR. **d.** Transfected cells with lentiviral shRNA containing mCherry were confirmed by immunofluorescence. **e.** Blood glucose levels of mice were checked weekly. Conditions for each experimental group (6 per each group) are described in the bottom panel. **f.** Body weights of mice. **g.** Bioluminescence images of organs excised from mice were taken, related to Figure 8h. *, $P < 0.05$; *n.s.*, not significant.

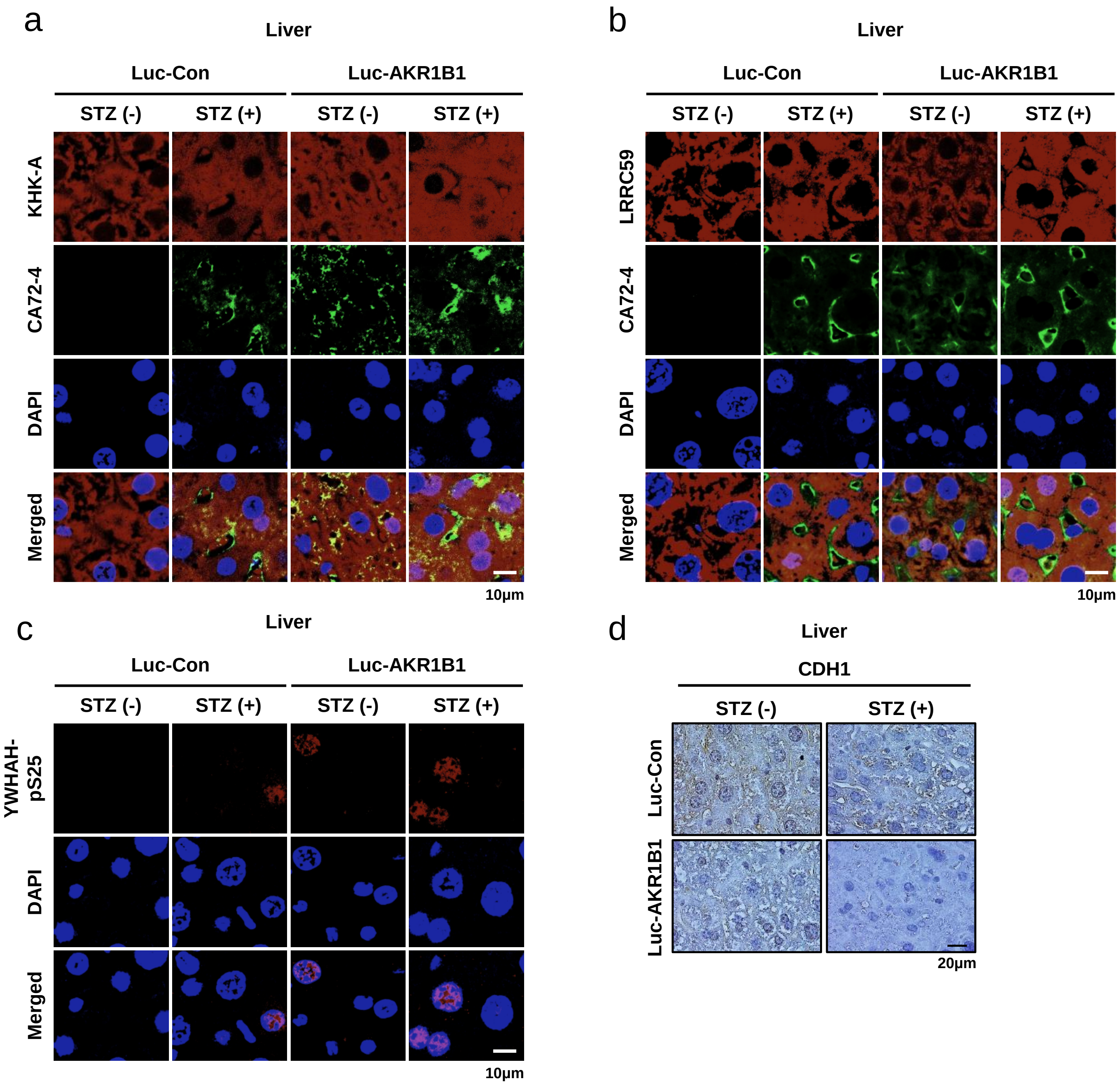

Supplementary Figure 20.

a-b. Representative immunofluorescence images related with figure 8j. The livers were stained with the antibodies against KHK-A (**a**) or LRRC59 (**b**), and anti-CA 72-4 antibody. KHK-A, LRRC59 were examined by Alexa Flour 594-conjugated secondary antibodies (red), and CA 72-4 were visualized by Alexa Flour 488-conjugated secondary antibodies (green). DAPI (blue) were stained to visualize nuclei. **c.** Representative immunofluorescence photographs related with figure 8k. YWHAH-pS25 and nucleus were stained with Alexa Flour 594-conjugated secondary antibodies (red) and DAPI (blue), and visualized under a fluorescence microscope. **d.** The liver tissues were immunostained with the antibody against CDH1. Representative pictures were related with figure 8l.

Supplementary Table 1. Antibodies

Antibodies	Company	Catalog No.	Dilution
FLAG	Sigma-Aldrich (St. Louis, MO)	F7425	1:1000
AKR1B1	Invitrogen (Carlsbad, CA)	PA5-29718	1:5000
HRP-conjugated goat anti-rabbit	Invitrogen (Carlsbad, CA)	G21234	1:5000
HRP-conjugated goat anti-mouse	Invitrogen (Carlsbad, CA)	G21040	1:5000
SORD	Novus Biologicals (Littleton, CO)	NBP1-87416	1:1000
LRRC59	Novus Biologicals (Littleton, CO)	NBP1-93953	1:1000
12-lipoxygenase	Novus Biologicals (Littleton, CO)	NBP1-90338	1:1000
KHK	Santa Cruz Biotechnology (Santa Cruz, CA)	sc-377411	1:1000
Slug	Santa Cruz Biotechnology (Santa Cruz, CA)	sc-166476	1:1000
N-cadherin	Santa Cruz Biotechnology (Santa Cruz, CA)	sc-7939	1:1000
Vimentin	Santa Cruz Biotechnology (Santa Cruz, CA)	sc-7558	1:1000
β-tubulin	Santa Cruz Biotechnology (Santa Cruz, CA)	sc-9104	1:1000
Aldolase B	Santa Cruz Biotechnology (Santa Cruz, CA)	sc-393278	1:1000
TWIST	Abcam (Cambridge, UK)	ab50581	1:1000
Luciferase	Abcam (Cambridge, UK)	ab21176	1:1000
Snail	Abcam (Cambridge, UK)	ab53519	1:1000
YWHAH	Cell Signaling Technology (Danvers, MA)	9640	1:1000
Lamin-B1	Cell Signaling Technology (Danvers, MA)	12586	1:1000
HA-tag	Cell Signaling Technology (Danvers, MA)	3724	1:1000
Myc-tag	Cell Signaling Technology (Danvers, MA)	2278	1:1000
CA 72-4	Origene (Rockville, MD)	CF190082	1:1000
KHK-A	Signalway Antibody LLC (Pearland, TX)	21708-2	1:1000
KHK-C	Signalway Antibody LLC (Pearland, TX)	21709-2	1:1000
His(6)-tag	MBL (Nagoya, Japan)	PM032	1:1000
HRP-conjugated rabbit anti-goat	Thermo Fisher Scientific (Waltham, MA)	81-4620	1:5000
E-cadherin	Thermo Fisher Scientific (Waltham, MA)	131700	1:1000
S25-phosphorylated YWHAH	Bioneer (Daejoen, South Korea)	-	-

Supplementary Table 2. Nucleotide sequences of siRNAs

siRNA		Sequence
Pan	Control	5'- AUGAACGUGAAUUGCUCAATT -3'
human	AKR1B1	5'- AGGAAUUUUUCCCAUUGGAUGAGTC -3’
human	SORD	5'- GGAUAUCAAGGGCGUGUUUCGAUAC -3’
human	KHK-A	5'- GUCAUCAUCAACGAGGCCAGUGGTA -3’
human	LRRC59	5'- AAGCUAGACCUGAGUAAGAACAAGC -3'
human	ALDOB	5'- CUUGCUAGUCAUUGGAAUCAAGCCG -3'
human	ALOX12	5'- GAUCCAGUAUCACUUGCUGAACACT -3'
human	YWHAH (3’ UTR)	5'- GUUUUGGAAUUCAAUGGGUAAAUAA -3'

Supplementary Table 3. Primers used in real-time quantitative PCR

Gene		Forward	Reverse
human	KHK-A	5'- TCATGGAAGAGAAGCAGATC -3'	5'- GGAGGTCATCCAGGACAAAA -3'
human	KHK-C	5'- TCATGGAAGAGAAGCAGATC -3'	5'- TGAAGTCGGCCACCAGGAAG -3'
human	CDH1	5'- TCTGGATAGAGAACGCATTG -3'	5'- TGTTGTCATTCTGATCGGTT -3'
human	GAPDH	5'- GAGTCAACGGATTTGGTCGT -3'	5'- TTGATTTTGGAGGGATCTCG -3'

Supplementary Table 4. Clinical information on gastric cancer patients

No.	Age	Sex	Organ	Diagnosis	pTNM	Stage	Follow-up months	Follow-up result	Cause of death
1	40	M	Stomach	adenocarcinoma, poorly differentiated	T4aN3aM1	IV	4	dead	cancer
2	43	F	Stomach	adenocarcinoma, poorly differentiated	T4aN0M0	II B	132	alive	.
3	61	F	Stomach	adenocarcinoma, moderately differentiated	T3N3aM0	III B	7	dead	cancer
4	45	F	Stomach	signet ring cell carcinoma	T3N3aM0	III B	132	alive	.
5	67	M	Stomach	adenocarcinoma, poorly differentiated	T3N1M0	II B	100	dead	chronic renal failure
6	53	F	Stomach	adenocarcinoma, poorly differentiated	T3N3aM0	III B	16	dead	cancer
7	67	M	Stomach	adenocarcinoma, moderately differentiated	T4aN3bM0	III C	13	dead	cancer
8	72	M	Stomach	adenocarcinoma, poorly differentiated	T3N3aM0	III B	1	dead	liver cirrhosis
9	61	M	Stomach	mucinous adenocarcinoma	T1bN2M0	II A	123	dead	pneumonia
10	65	M	Stomach	adenocarcinoma, poorly differentiated	T4aN0M0	II B	131	alive	.
11	70	M	Stomach	adenocarcinoma, moderately differentiated	T4aN2M0	III B	9	dead	cancer
12	62	M	Stomach	adenocarcinoma, well differentiated	T3N2M0	III A	45	dead	cancer
13	62	F	Stomach	signet ring cell carcinoma	T3N2M0	III A	26	dead	cancer
14	52	M	Stomach	papillary adenocarcinoma	T2N0M0	I B	131	alive	.
15	60	M	Stomach	adenocarcinoma, poorly differentiated	T3N3aM1	IV	6	dead	cancer
16	66	M	Stomach	adenocarcinoma, poorly differentiated	T4aN3bM0	III C	6	dead	cancer
17	41	M	Stomach	signet ring cell carcinoma	T3N3aM0	III B	131	alive	.
18	60	F	Stomach	adenocarcinoma, poorly differentiated	T3N3aM0	III B	131	alive	.
19	59	M	Stomach	adenocarcinoma, poorly differentiated	T4aN3bM0	III C	36	dead	cancer
20	62	M	Stomach	adenocarcinoma, poorly differentiated	T3N3bM0	III B	41	dead	cancer
21	49	M	Stomach	adenocarcinoma, poorly differentiated	T4bN3bM0	III C	4	dead	cancer
22	64	M	Stomach	mucinous adenocarcinoma	T3N1M0	II B	83	alive	.
23	55	M	Stomach	undifferentiated carcinoma	T4aN3bM0	III C	7	dead	cancer
24	56	F	Stomach	adenocarcinoma, poorly differentiated	T3N2M0	III A	36	dead	cancer
25	48	M	Stomach	adenocarcinoma, poorly differentiated	T3N2M0	III A	10	dead	cancer
26	65	F	Stomach	mucinous adenocarcinoma	T4aN3aM0	III C	15	dead	cancer
27	63	M	Stomach	adenocarcinoma, well differentiated	T3N2M0	III A	130	alive	.
28	60	M	Stomach	adenocarcinoma, well differentiated	T3N0M0	II A	101	dead	cancer
29	42	M	Stomach	signet ring cell carcinoma	T3N1M0	II B	130	alive	.
30	55	M	Stomach	mucinous adenocarcinoma	T3N3aM0	III B	14	dead	cancer
31	58	M	Stomach	adenocarcinoma, moderately differentiated	T3N3bM0	III B	10	dead	cancer
32	72	F	Stomach	adenocarcinoma, moderately differentiated	T1bN2M0	II A	15	dead	cancer
33	53	M	Stomach	adenocarcinoma, poorly differentiated	T3N0M0	II A	130	alive	.
34	46	M	Stomach	adenocarcinoma, poorly differentiated	T4bN0M0	III B	7	dead	cancer
35	64	M	Stomach	adenocarcinoma, moderately differentiated	T2N1M0	II A	130	alive	.
36	65	M	Stomach	adenocarcinoma, poorly differentiated	T2N1M0	II A	68	dead	cancer
37	45	F	Stomach	adenocarcinoma, poorly differentiated	T2N1M0	II A	129	alive	.
38	58	F	Stomach	adenocarcinoma, well differentiated	T3N0M0	II A	2	dead	cancer
39	68	F	Stomach	adenocarcinoma, poorly differentiated	T3N3aM1	IV	5	dead	cancer
40	66	M	Stomach	adenocarcinoma, poorly differentiated	T4aN3aM0	III C	6	dead	cancer
41	63	M	Stomach	adenocarcinoma, poorly differentiated	T3N0M0	II A	81	dead	cancer
42	52	F	Stomach	adenocarcinoma, moderately differentiated	T3N2M0	III A	11	dead	cancer
43	35	F	Stomach	adenocarcinoma, poorly differentiated	T1aN0M0	I A	132	alive	.
44	44	F	Stomach	adenocarcinoma, well differentiated	T1bN0M0	I A	132	alive	.
45	55	M	Stomach	adenocarcinoma, well differentiated	T1aN0M0	I A	132	alive	.
46	40	M	Stomach	signet ring cell carcinoma	T1aN0M0	I A	131	alive	.
47	66	M	Stomach	adenocarcinoma, moderately differentiated	T1bN0M0	I A	131	alive	.
48	65	F	Stomach	adenocarcinoma, moderately differentiated	T1bN0M0	I A	3	dead	chronic renal failure
49	62	F	Stomach	adenocarcinoma, poorly differentiated	T1bN0M0	I A	130	alive	.
50	60	F	Stomach	adenocarcinoma, moderately differentiated	T1bN0M0	I A	130	alive	.
51	70	F	Stomach	adenocarcinoma, moderately differentiated	T1bN1M0	I B	130	alive	.
52	52	M	Stomach	adenocarcinoma, well differentiated	T1bN0M0	I A	130	alive	.
53	40	M	Stomach	signet ring cell carcinoma	T1bN1M0	I B	130	alive	.
54	34	F	Stomach	signet ring cell carcinoma	T1aN1M0	I B	130	alive	.
55	60	M	Stomach	adenocarcinoma, well differentiated	T1bN0M0	I A	130	alive	.
56	68	M	Stomach	adenocarcinoma, well differentiated	T1bN1M0	I B	129	alive	.
57	52	M	Stomach	adenocarcinoma, well differentiated	T2N0M0	I B	129	alive	.
58	62	M	Stomach	signet ring cell carcinoma	T1aN0M0	I A	129	alive	.
59	60	M	Stomach	adenocarcinoma, well differentiated	T2N0M0	I B	99	dead	cancer
